# Probing the sequence constraints for the stable incorporation of chimeric NSP1 segments into infectious rotaviruses

**DOI:** 10.1099/jgv.0.002275

**Published:** 2026-07-03

**Authors:** Joseph Kendra, Gabriel I. Parra

**Affiliations:** 1 Division of Viral Products, Center for Biologics Evaluation and Research, Food and Drug Administration, Silver Spring, Maryland, USA

**Keywords:** NSP1, reverse-genetics, rotavirus

## Abstract

Rotaviruses are a global cause of acute paediatric gastroenteritis. They present a genome of 11 dsRNA segments that have been successfully incorporated into a plasmid-based reverse-genetics system that enables the generation of reassortant and chimeric viruses as a possible foundation for multi-virus vaccines. Studies have shown that these chimeric segments are pruned across successive passages, highlighting a need to better define the constraints that govern segment recognition and stable incorporation into mature viruses. This work leveraged the reverse-genetics system to investigate these constraints by introducing a series of modifications to a chimeric NSP1 sequence with an internally cloned GFP insert (NSP1gfp). Across ten serial passages, a pruned NSP1gfp segment emerged and eventually outcompeted the original construct, resulting in a loss of GFP expression. The removal of the entire NSP1 ORF sequence 3′ of the insert resulted in prolonged GFP expression, but closer examination of rotavirus RNA suggested that these modifications were merely delaying replacement from pruned segments, rather than avoiding their emergence. A series of systematic truncations to the remaining NSP1 ORF sequence 5′ of the GFP insert showed that as little as 27 nt of the original coding sequence is needed for rescue of infectious chimeric rotavirus. Despite a near-total replacement of NSP1 with GFP, pruned segments emerged and were preferentially incorporated into later passages, which was correlated with increased infectivity at the expense of GFP expression. These results suggest that factors beyond excess segment size drive pruning of chimeric sequences. Closer sequence analysis of pruned segments revealed that pruning occurred at high GC regions of the insert, suggesting an inherent incompatibility with the genome composition of rotavirus segments. These findings shed light on the requirements for rotavirus segment recognition and stability, as well as provide important considerations for the usage of this model as a potential vaccine platform.

## Data Availability

Sequence information from the viral passages was submitted to the Sequence Read Archive (BioProject accession number: PRJNA1463220).

## Introduction

Group A rotaviruses (RVs) are a major worldwide cause of acute gastroenteritis and dehydrating diarrhoea in children under the age of 5 years [1]. Although several effective vaccines against rotavirus have been developed and employed to great success, the disease still accounts for 155,000–200,000 deaths annually, and the current vaccines present diminished effectiveness against predominant rotavirus strains of certain geographic regions [2,4]. Thus, there remains a continued need for the development of innovative new rotavirus vaccines. Rotaviruses present a genome of 11 segments of dsRNA, which have been successfully integrated into T7 plasmids, using the genome from the simian prototype rotavirus, SA11 [5,6]. This plasmid-based system can be transfected *en masse* into T7-permissible cell lines to express RV proteins and eventual assembly of mature recombinant rotavirus (rRV) particles, which are then amplified via co-culture of MA104 cells. Rescued rRVs are fully infectious and demonstrate replication kinetics similar to their wild-type counterparts [5,7]. The modular nature of the reverse-genetics system enables the direct manipulation of segments used in the generation of rRVs, allowing for in-depth investigations of recombinant or chimeric viruses [8,12].

Previous studies have demonstrated that rRVs can tolerate direct insertions of foreign gene sequences into the sequence of NSP1, an antagonist of host cell innate immunity that is non-essential to mature RV propagation in cell culture systems [13,15]. This has allowed for the integration of fluorescent proteins into rRV with detectable expression in infected cells and lays the foundation for the development of chimeric segments of critical viral antigens [7,16]. However, while viral progeny containing this chimeric segment can successfully be rescued from transfections, substantial pruning to the insert sequence is reported across serial passages, leading to the eventual ablation of insert functionality [16]. The mechanism that drives this pruning is unknown, but it has been further demonstrated that segment integrity can be stabilized across ten passages through deletions to the defunct NSP1 ORF 3′ of the insert sequence, suggesting that excess length is a potential factor for long-term segment instability [7,16]. This strategy has been further refined by the inclusion of a 2A self-cleaving peptide upstream of the internal insert [7] or at the 3′ terminal end of whole NSP1 or NSP3 ORFs to which the chimeric sequence can be appended [17,20]. This approach has been shown to stabilize the expression of chimeric sequences across serial passages and has been leveraged both as a fluorescent tool for neutralization assays [20] and to express antigens from viruses like norovirus and SARS-CoV-2 as a candidate vaccine platform [21,23]. Despite these advances, further investigation into the original insertion model may assist in resolving the constraints that govern rotavirus segment stability. As neither the insert pruning nor the targeted 3′ truncations for stability restore the original function of NSP1 in rRVs [7,16], the additional question arises regarding the minimum required original sequence needed to successfully introduce a chimeric segment into infectious rRVs.

In this study, we used reverse genetics to generate a series of systematically truncated chimeric NSP1 sequences to interrogate the minimum required original sequence needed for efficient replication and incorporation into rRVs. We found that viable rRVs expressing GFP that contain only the first 81 nt of the NSP1 ORF 5′ of the chimeric insert can be rescued and successfully passaged ten times. Additional truncation experiments on that NSP1 5′ ORF region demonstrated that as little as 27 nt of the original sequence is needed for rescue of rRVs, allowing for a near-complete substitution of NSP1 with a foreign reporter gene. However, results indicate that these modifications merely delay the emergence of pruned segments that eventually outcompete the original construct, which in all cases was correlated with a loss of insert sequence function and improved rRV fitness. The specific mechanisms of this pruning remain unclear, but results strongly suggest that gene composition rather than excess segment size is the driver for sequence excision.

## Methods

### Cell culture

Baby hamster kidney BHK/T7-9 fibroblasts (kindly provided by Ursula Bucholz [24]) were cultured in Glasgow’s Minimal Essential Medium (G-MEM) (Gibco) supplemented with 10% FBS (Gibco), 5% l-glutamine and 10% essential amino acids. BHK/T7-9 cell cultures were maintained through passages with alternating presence of 250 µg ml^−1^ geneticin (Gibco). Monkey kidney epithelial MA104 cells (ATCC) were grown in Eagle’s Minimum Essential Medium (EMEM) (Gibco) supplemented with 10% FBS (Gibco).

### rRV plasmids

The original set of rRV rescue plasmids (pT7-VP1SA11, pT7-VP2SA11, pT7-VP3SA11, pT7-VP4SA11, pT7-VP6SA11, pT7-VP7SA11, pT7-NSP1SA11, pT7-NSP2SA11, pT7-NSP3SA11, pT7-NSP4SA11 and pT7-NSP5SA11), as well as support plasmids encoding the FAST gene derived from Nelson Bay orthoreovirus strain Miyazaki-Bali/2007 (accession no. AB9082824) (pCAG-FAST-p10) and Vaccinia virus capping enzymes D1R (accession no. NC006998) (pCAG-D1R) and D12L (accession no. NC006998) (pCAG-D12L), was kindly provided by the Takeshi Kobayashi laboratory group through Addgene [5]. Complete plasmid maps of all plasmids used in the original publication are compiled on the Addgene webpage (https://www.addgene.org/browse/article/25158/). For the construction of SA11_NSP1gfp, the pT7-NSP1SA11 plasmid was linearized at the 81 nt of the NSP1 ORF (FWD: 5′-TCTATTTGAATGCCAGTTCC-3′, REV: 5′-ATTTGCACCAATGTTGCATAATCTCCG-3′). The sequence for GFP (accession no. MN968806) was cloned into this linearized plasmid via the In-Fusion HD EcoDry Cloning kit (TakaraBio). Additional primers (FWD: 5′-TTGCGCGATCCGTGATATGTAT-3′, REV: 5′-TCAATTTTACTAAGAAAATATTTTATATTACACCATTTACAG-3′) and the Rapid DNA Dephos and Ligation kit (Roche) were used to develop an alternative NSP1gfp plasmid with a silently mutated BamHI restriction site downstream of the GFP insertion sequence (NSP1gfp_BamHI_). The presence of the mutation in the plasmid and in amplified viral DNA products was confirmed via digestion with BamHI enzymes at 37 °C for 2 h (New England Biolabs). Truncated NSP1gfp plasmids were constructed by linearizing pT7-NSP1SA11 at the 5′ and 3′ UTR sequences of NSP1 (FWD: 5′-AATTATGTCACTATCTAATTATACAG-3′, REV: 5′-CATGGCTAACACAAGACTTTTCAAAAAAAAGCC-3′) and cloning in the modified SA11_NSP1gfp sequences via the In-Fusion Cloning kit. The construction of plasmids containing incrementally truncated SA11_NSP1gfp sequences was synthesized from Genscript. All insert sequences were verified via capillary sequencing using primers that map to the pT7 plasmid backbone (FWD: 5′-CTGTGGATAACCGTATTACCG-3′, REV: 5′-GCTAGTTATTGCTCAGCGG-3′). Modified segment sequences are presented in Supplemental Text (available in the online Supplementary Material), and plasmids are available upon request.

### Transfection, recovery and passaging of rRVs

BHK/T7-9 cells were seeded (2×10^5^) into 6-well plates (Celltreat) and allowed to grow until monolayers of 80–90% confluency were achieved. Media were then replaced with FBS-free G-MEM, and cells were transfected with rRV plasmids prepared in 250 µl Opti-MEM (Gibco) and 2 µl of TransIT-LT1 transfection reagent (Mirus Bio) per µg of plasmid DNA. Plasmid concentrations of the rRV system were prepared as follows: 0.8 µg of plasmids pT7-VP1SA11, pT7-VP2SA11, pT7-VP3SA11, pT7-VP4SA11, pT7-VP6SA11, pT7-VP7SA11, pT7-NSP1SA11 (or applicable NSP1gfp variant), pT7-NSP3SA11, pT7-NSP4SA11, pCAG-D1R and pCAG-D12L; 2.0 µg of plasmids pT7-NSP2SA11 and pT7-NSP5SA11; and 0.015 µg of pCAG-FAST. Following a 2-day incubation at 37 °C, transfected BHK/T7-9 cells were co-cultured with MA104 cells (2×10^5^) and incubated an additional 3 days at 37 °C in FBS-free EMEM supplemented with 0.5 µg ml^−1^ trypsin (Gibco). rRVs were rescued by lysing transfected cells with three cycles of freeze/thaw and centrifugation of residual cell debris. Infection of rRVs into fresh monolayers of MA104 cells was carried out as a blind passage, in which 150 µl of recovered virus lysate was brought to a concentration of 10 µg ml^−1^ trypsin in FBS-free EMEM. The trypsin-activated lysate was added to confluent MA104 cells in 6-well plates and adsorbed for 1 h at 37 °C. Adsorbed MA104 cells were then washed and cultured in FBS-free EMEM with 0.5 µg ml^−1^ trypsin and incubated at 37 °C for 7 days or until lysis by cytopathic effects (CPEs) was observed. rRV passages were harvested and serially passaged as described until P10 or recovery failure.

### Quantification of rRV passages

The TCID_50_ was calculated for the blind serial passages of rRVs for WT SA11 and viruses containing a modified NSP1 segment. Lysates of each rRV passage were infected in triplicate in 96-well plates of MA104 cells as 10-fold dilutions in FBS-free EMEM with 0.5 µg ml^−1^ trypsin beginning at 10^−2^. Infected cells were incubated at 37 °C for 7 days and monitored for CPE. Cells were then fixed with chilled methanol (Sigma-Aldrich), stained with 0.1% crystal violet (Thermo Scientific), and TCID_50_ ml^−1^ quantitation for each passage of each rRV was conducted using the Spearman Kärber method (via calculation template sheet described elsewhere) [25]. For replication kinetics assays, MA104 cells were infected with P2, P3, P9 and P10 rRV stocks at 0.001 IU per cell. Infection time points were taken at 0, 24, 48 and 72 hours post-infection (HPI) for each virus and passage, and their respective TCID_50_ ml^−1^ titres were set up and calculated as described above. Data and statistics were plotted using Prism v11.

### Microscopy of rRV-infected cells

96-well plates (CellTreat) of confluent MA104 cells were infected with each respective rRV passage at 0.001 IU per cell to normalize infectivity and allow for observation of infected cells before complete monolayer disruption through CPE. Brightfield and fluorescent images of infected cells were collected at 24 HPI via the BZ-X810 All-in-One Fluorescence Microscope (Keyence). Infected cells were then fixed with chilled methanol for counterstaining. Rotavirus antigens were detected with 1 : 2,000 anti-SA11 polyclonal rabbit sera (kindly provided by the Kristen Ogden laboratory group), followed by 1 : 500 AlexaFluor 568 goat anti-rabbit IgG FC secondary antibodies (Invitrogen). Host cell nuclei were detected by a 1 : 1,000 stain of DAPI (Thermo Fisher). Images for stained cells were collected via fluorescent microscopy as described above. Quantification of GFP expression was conducted via BZ-II Analyzer (Keyence) and plotted using Prism v11.

### Rotavirus genome and modified NSP1 segment analysis

Rotavirus genomes were extracted from lysates treated with RNase A (New England Biolabs) and harvested via the QIAamp Viral RNA Mini Kit (QIAGEN). The electropherotypes for each virus genome were visualized via 6% TBE gel with Sybr Safe DNA gel stain (Invitrogen). Amplified DNA for the NSP1 segment for WT SA11, NSP1gfp and truncated variants was generated via the SuperScript III One-Step RT-PCR System (Invitrogen) using primers that universally map to the UTRs of all NSP1 segments (F: 5′-GGCTTTTTTTTGAAAAGTCTTGTGTTAGC-3′, R: 5′-CCAGCTAGGCGCTACTCTAGTC-3′) and visualized via 1% agarose gel with ethidium bromide (BioRad). For sequence analysis of modified NSP1 segments, DNA amplicons were purified from agarose gels via QIAquick Gel Extraction Kit (QIAGEN) and quantified via Qubit 3 Fluorometer (Invitrogen). Next-generation sequencing (NGS) was then performed via MiSeq Reagent Nano kit v2 (Illumina). Consensus sequences and viral population analyses based on NGS were performed using the HIVE environment [26]. Clonal population analysis was employed to identify haplotypes indicative of virus subpopulations, adapted from previous studies [27]. Consensus sequences of NSP1 construct mRNAs from passages P1 and P10 were aligned with mega7 and submitted to RNAfold (ViennaRNA Web Services, http://rna.tbi.univie.ac.at [28]) for the generation of secondary structure maps and minimum free energy analyses. Secondary structure maps were annotated in Affinity Designer (Serif Europe Ltd.) to highlight inserted GFP sequences. The GC content of NSP1 constructs was evaluated and visually mapped via RStudio using the seqinr package [29].

## Results

### Generation of rotaviruses with reduced sequence NSP1gfp segments

**Fig. 1.**
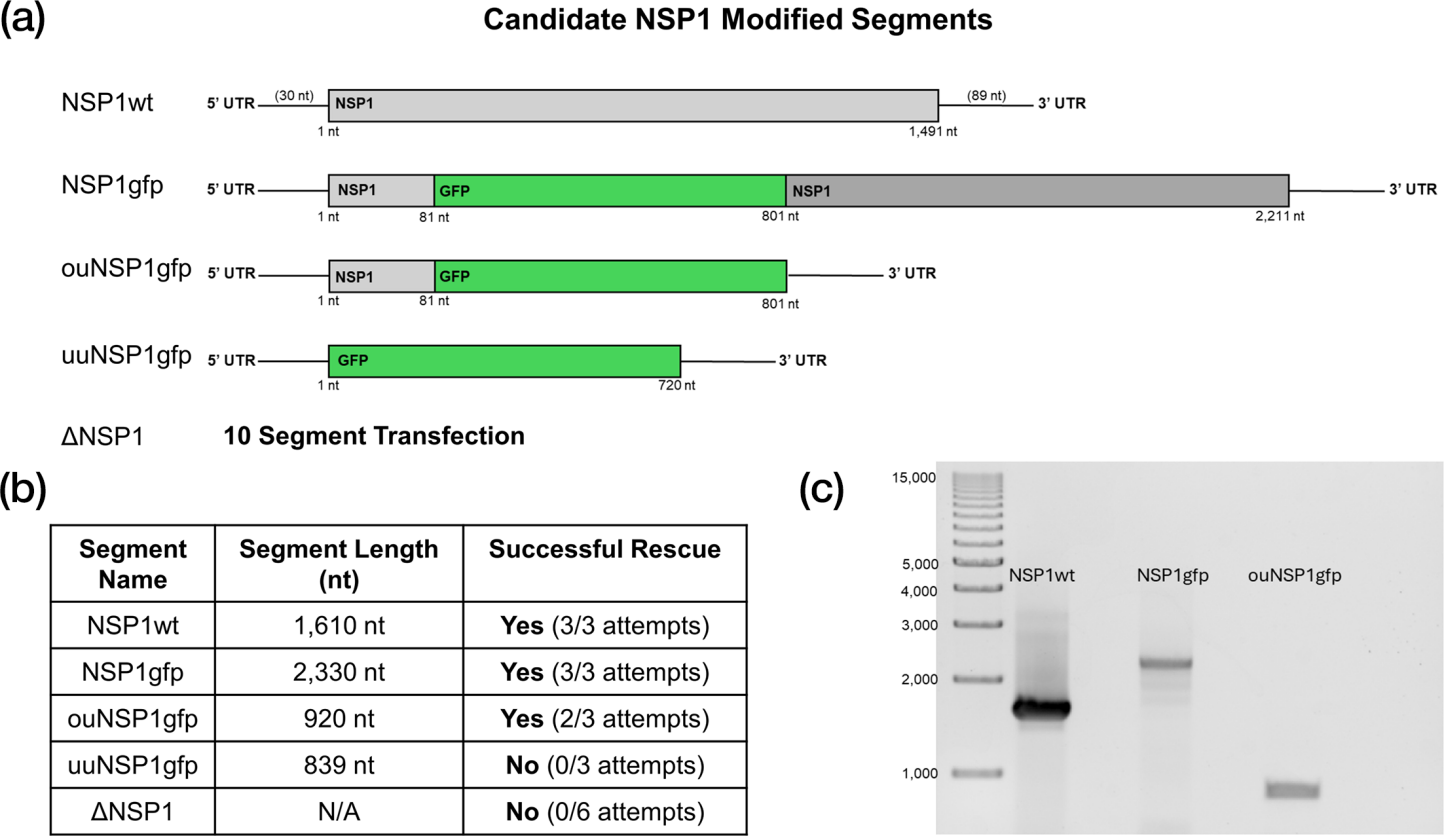
Schematics and rescue efficiency of modified NSP1 segments. (**a**) The following modifications were introduced into the NSP1 sequence for SA11 rotavirus (NSP1wt) for use in the reverse-genetics system: (i) a GFP reporter sequence cloned 81 nt into the NSP1 ORF that retains the remaining NSP1 sequence 3′ of the insertion (NSP1gfp), (ii) a modification of NSP1gfp that retains the ORF sequence 5′ of the insert but removes all downstream sequence save for the 3′ UTR (ouNSP1gfp), (iii) the GFP sequence flanked by the 5′ and 3′ NSP1 UTRs (uuNSP1gfp) and (iv) an attempted SA11 reverse-genetics transfection in the absence of an NSP1 segment (ΔNSP1). Segment UTRs are denoted with solid black lines, NSP1 ORF regions are denoted with light grey bars, the GFP insert sequence is represented with a light green bar and out-of-frame ORF sequences downstream of the insert are shown with a dark grey bar. Numbers denote UTR lengths and nt markers for points of interest along the NSP1 ORF. (**b**) Modified NSP1 segments are presented with their respective lengths compared to the full NSP1wt sequence (1,610 nt). Successful rescue of rRVs with respective NSP1 modifications is reported, alongside the number of rescue attempts for each rRV. (**c**) Reverse-transcription PCR was performed on RNA isolated from P1 passages of WT NSP1, NSP1gfp and ouNSP1gfp to better visualize the change in segment size resulting from chimeric modifications. The resulting DNA amplicons for NSP1wt, NSP1gfp and ouNSP1gfp are shown on an agarose gel.

We utilized the SA11 reverse-genetics system to test several candidate NSP1gfp segments for rRV incorporation and rescue. The sequence for GFP was inserted into the NSP1 coding region as previously depicted for other reporter proteins (NSP1gfp) [16]. To broadly identify the NSP1 regions necessary for segment recognition and encapsidation, variations of NSP1gfp were made as follows: a segment that retains the 81 nt of SA11 ORF sequence 5′ of the GFP insert but excises all but the UTR downstream of the insert sequence (ouNSP1gfp) and a segment comprised of only the GFP sequence flanked by the NSP1 WT UTRs (uuNSP1gfp). Additionally, to verify the necessity of the presence of the NSP1 segment for the rescue of mature rRVs, a reverse-genetics transfection was attempted in the absence of an NSP1 plasmid (ΔNSP1). Schematics of these modified NSP1 segments compared to the wild-type (NSP1wt) are presented in Fig. 1a.

The outcome of reverse genetics transfections with modified NSP1 segments is summarized in Fig. 1b. Consistent with other investigations, transfections with the NSP1gfp segment resulted in the rescue of chimeric rRVs that were successfully passaged up to ten times in MA104 cells. Transfections with the ouNSP1gfp variation also resulted in successful rescue and serial passage of mature rotavirus. Isolation of NSP1 segments from the first passage of rescued rRVs via reverse-transcription PCR using primers specific to the NSP1 UTRs showed respective size differences of NSP1gfp and ouNSP1gfp segments relative to NSP1wt (Fig. 1c). The full-sequence length of the NSP1wt segment is 1,610 nt, but the inclusion of the GFP insert (720 nt) in NSP1gfp increases the length to 2,330 nt. In contrast, the removal of the out-of-frame NSP1 ORF 3′ of the GFP insert produces a much smaller ouNSP1gfp segment of 920 nt. To check for the residual presence of reverse-genetics plasmids, rRV stocks were additionally tested via PCR using primers specific to the pT7 backbone. Residual plasmids from the reverse-genetics transfection were not detected in any of the virus stocks, confirming that recovered NSP1 RNA is the product of rRVs (Fig. S1). rRVs were unable to be recovered from uuNSP1gfp or ΔNSP1 transfections (Fig. 1b, 0/3 and 0/6 attempts, respectively), suggesting that the presence of NSP1 and at least some portion of the initial ORF sequence is required for incorporation of GFP-modified segments into mature rotavirus particles.

### Increased segment fidelity and function of ouNSP1gfp

**Fig. 2.**
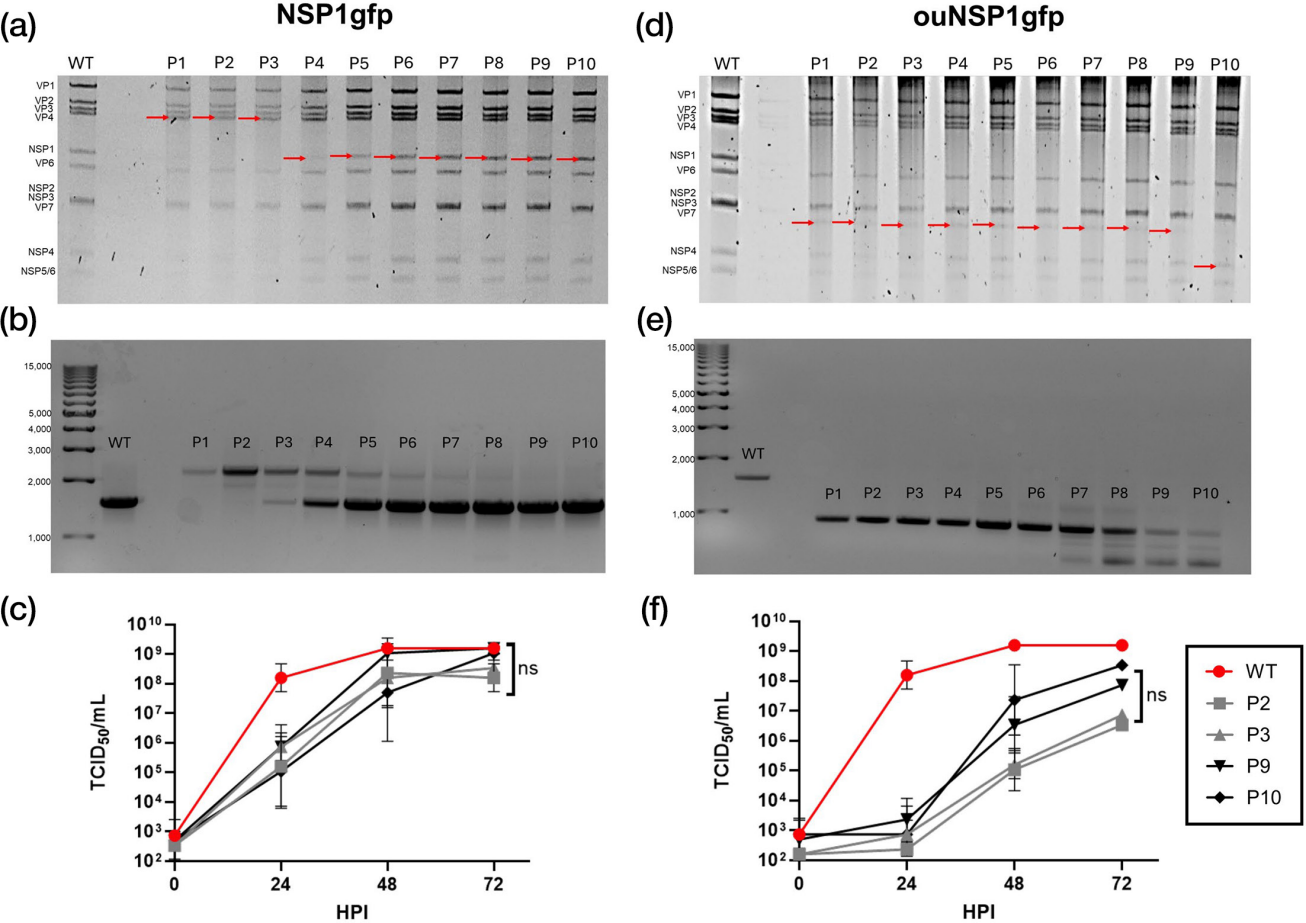
Evaluation of modified NSP1 segment stability across serial passages. (**a**) PAGE analysis of SA11 NSP1gfp rRV genome segments across ten serial passages compared to the NSP1wt electropherotype. The size of the NSP1gfp segment across serial passages is shown with red arrows. (**b**) Agarose gel of amplified NSP1 segment DNA from NSP1wt and NSP1gfp rRVs across ten serial passages. (**c**) Replication kinetics of rRVs containing unpruned (grey: P2, P3) or pruned (black: P9, P10) NSP1gfp segments alongside an NSP1wt control (red). Time points were analysed as two biological replicates of technical triplicates and shown as TCID_50_ ml^−1^. Titres of unpruned and pruned groups were compared via Mann–Whitney *U* test (‘n.s.’=not significant). (**d**) PAGE analysis of SA11 ouNSP1 rRV genome segments across ten serial passages compared to NSP1wt electropherotype. The size of the ouNSP1gfp segment across serial passages is tracked with red arrows. (**f**) Agarose gel of amplified NSP1 segment DNA from NSP1wt and ouNSP1 rRVs across ten serial passages. (**g**) Replication kinetics of rRVs containing unpruned (grey: P2, P3) or pruned (black: P9, P10) ouNSP1gfp segments alongside a NSP1wt control (red). Time points were analysed as two biological replicates of technical triplicates and shown as TCID_50_ ml^−1^. Titres of unpruned and pruned groups were compared via the Mann–Whitney *U* test (‘n.s.’, not significant).

Following the successful rescue of rRVs containing either an NSP1gfp or ouNSP1gfp segment, we sought to investigate viral fitness and chimeric segment stability across serial passages. Electropherotype analysis of the NSP1gfp rRV genome showed a marked decrease in size of the chimeric segment to that of WT by passage P5 (Fig. 2a). These results are consistent with similar observations in the literature [16]. We next generated DNA amplicons of these NSP1gfp segments to provide closer analysis of this segment degradation. The resulting amplicons of the passaged NSP1gfp segments showed that the pruned NSP1gfp segment was observable as early as passage P2, where it proceeded to outcompete its larger intact counterpart, fully replacing it by P9 (Fig. 2b). Fainter species of bands were additionally observed between the full-length NSP1 and pruned segments at these passages, suggesting that multiple subpopulations of pruned segments are being generated or that multiple excision steps are required to reach the final size of the pruned variant. Replication kinetics for rRVs containing unpruned (P2, P3) or pruned (P9, P10) segments were virtually identical, with endpoint titres similar to those observed for NSP1wt (Fig. 2c).

It was notable that the pruned variant that overtook NSP1gfp appeared to be of identical size to the NSP1wt segment. To rule out the possibility that these results were the product of trace contamination from NSP1wt plasmids during initial transfection, we developed an alternative NSP1gfp plasmid with a BamHI restriction site silently mutated into the ORF sequence downstream of the GFP insert (NSP1gfp_BamHI_) (Fig. S2a). rRVs containing NSP1gfp_BamHI_ were successfully rescued and serially passaged. Similar to NSP1gfp, P10 NSP1gfp_BamHI_ rRVs presented a pruned variant of a similar size to NSP1wt, which interestingly had not fully outcompeted the larger original segment (Fig. S2b). Follow-up restriction digestion analysis with BamHI enzymes cleaved both P10 NSP1gfp_BamHI_ segments at the predicted location (Fig. S2c), confirming that both segments share a common origin. NGS analysis of the pruned segments revealed two subpopulations with the majority of the GFP insert excised alongside a small region of the immediately 3′ NSP1 ORF sequence (Fig. S2d), presenting similar pruning patterns observed elsewhere in the literature [16].

In contrast, electropherotype visualization of rRVs containing ouNSP1gfp segments were able to retain their expected size across all ten serial passages, albeit with a diminished band presence starting at passage P9 (Fig. 2d). Reverse-transcription PCR inspection of ouNSP1gfp DNA amplicons revealed the presence of a smaller band indicative of a pruned chimeric segment that was detectable in passage P7 and grew in intensity over the remaining passages (Fig. 2e). The bands of the original ouNSP1gfp segment correspondingly became fainter over these passages but were not surpassed by the pruned segment band until passage P9. A comparison of unpruned (P2, P3) and pruned (P9, P10) ouNSP1gfp replication kinetics demonstrated marginally increased replicative efficiency of rRVs containing pruned segments that did not reach statistical significance (Fig. 2f). These results suggest that, contrary to initial suspicions that pruning is a corrective measure against segments of excessive length, the engineering of shorter chimeric NSP1 sequences may only succeed in delaying the emergence and outcompetition of pruned sequences rather than circumventing them entirely.

### Chimeric segment pruning associated with a loss of insert functionality

**Fig. 3.**
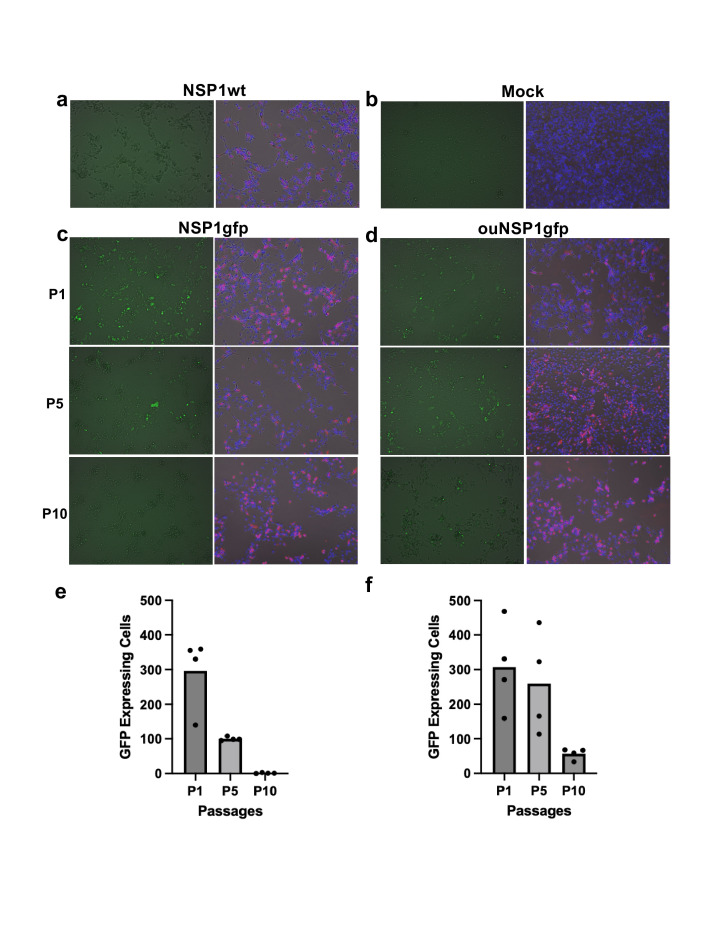
Evaluation of GFP expression in modified NSP1 rRVs across serial passages. (Left) Fluorescent microscopy images examining brightfield overlayed with GFP expression (green) of live-infected MA104 cells. (Right) Fluorescent microscopy images of methanol-fixed MA104 cells stained to detect host cell nuclei (DAPI, blue) and rotavirus antigen (rabbit anti-SA11 polyclonal sera and AlexaFluor 568 IgG FC, red). (**a**) MA104 cells infected with NSP1wt rRVs and (**b**) uninfected cells are presented as negative controls. Fluorescent microscopy images are provided for cells infected by (**c**) NSP1gfp and (**d**) ouNSP1gfp rRVs across ten serial passages. Passages P1, P5 and P10 are presented for both rRV lineages. Additional fluorescent images were taken for these samples at predetermined quadrants and used to quantify GFP expression across serial passages for (**e**) NSP1gfp and (**f**) ouNSP1gfp.

The insertion of a GFP reporter sequence into an NSP1wt segment results in the production of fluorescent proteins that are visible in rRV-infected cells. To assess the functionality of the inserted sequence across serial passages, we infected MA104 cells with passages P1, P5 and P10 of NSP1gfp or ouNSP1gfp rRVs. The fluorescence of infected cells was determined through microscopy analysis (Fig. 3). Compared to uninfected and NSP1wt-infected controls (Fig. 3a and b), the serial passages of rRVs containing the NSP1gfp segment presented robust expression of GFP in infected cells that decreased precipitously by passage P5 and was virtually undetectable by P10 (Fig. 3c and e). Consistent detection of SA11 rotavirus antigen was observed across all passages, suggesting that the observed decline in GFP expression was primarily caused by segment pruning (Fig. 3c). Interestingly, fluorescence was observed in passages where pruned NSP1gfp segments had outcompeted the original chimeric sequence, indicating that residual expression is either the result of full length NSP1gfp continuing to circulate at low levels or that the pruning process does not always fully ablate the function of the GFP insert sequence. In contrast, cells infected with ouNSP1gfp rRVs presented a slower decline of fluorescence across serial passages, and GFP expression was still visible at passage P10 (Fig. 3d and f). These results demonstrate improved stability and functionality of the inserted GFP sequence across serial passaging following the deletion of the downstream NSP1 ORF, consistent with the continued presence of non-degraded ouNSP1gfp segments at the end of serial passaging (Fig. 2e).

### Systematic truncations to the ouNSP1gfp segment demonstrate the minimum viable sequence of NSP1 5′ ORF

**Fig. 4.**
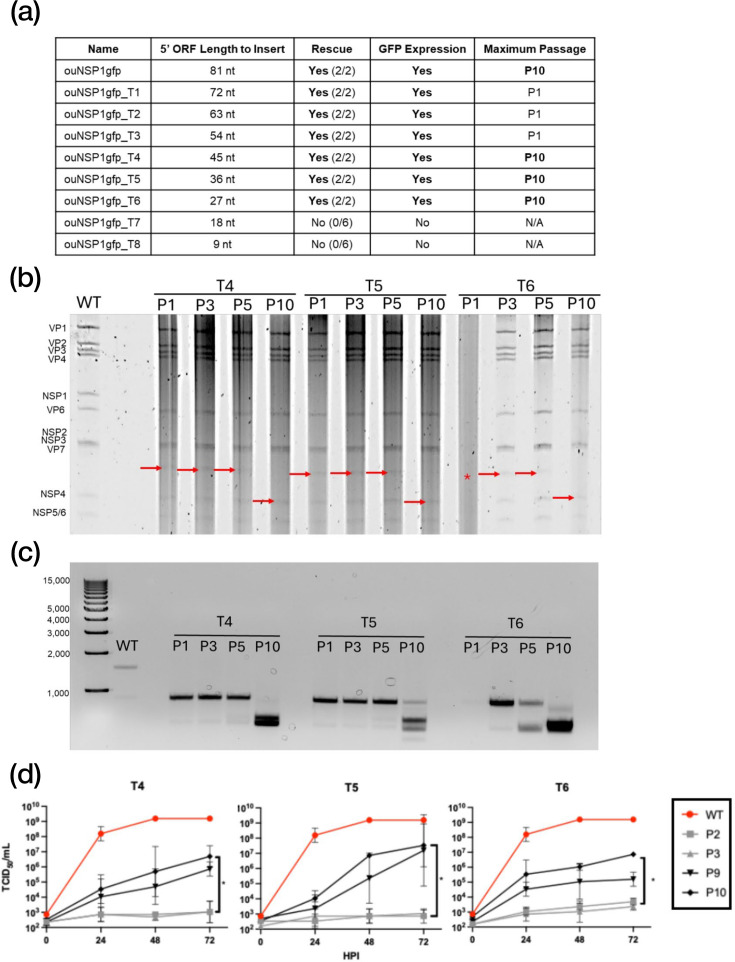
Evaluation of systematic truncations to NSP1 ORF 5′ of GFP insert. (**a**) Naming rationale and results table of the series of truncated segments derived from ouNSP1gfp. Each successive truncated segment in the series has the remaining SA11 ORF sequence reduced by 9 nt immediately 5′ of the GFP insert. The first truncation (ouNSP1gfp_T1) retains 72 nt of the WT ORF sequence from the original 81 nt of ouNSP1gfp, while the second truncation (ouNSP1gfp_T2) has been reduced to 63. A total of 8 truncated segments were developed (ouNSP1gfp_T1–T8). Following attempted transfection, the first passage of MA104 cells was monitored for up to 10 days for signs indicative of infectious rRVs (CPE, GFP expression). Successful rescue of rRVs’ respective truncated ouNSP1gfp segments is reported, alongside the number of rescue attempts for each rRV. (**b**) PAGE analysis of truncated ouNSP1gfp rRV genome segments across ten serial passages compared to the NSP1wt electropherotype. Passages P1, P3, P5 and P10 are presented for each truncated ouNSP1gfp rRV. The size of the truncated segments across serial passages is shown with red arrows. The red * in the P1 lane for ouNSP1gfp_T6 denotes an inability to extract sufficient viral RNA due to poor initial titres. (**c**) Agarose gel of amplified NSP1 segment DNA from WT SA11 and truncated ouNSP1 rRVs across ten serial passages. (**d**) Replication kinetics of rRVs containing unpruned (grey: P2, P3) or pruned (black: P9, P10) truncated ouNSP1gfp segments alongside a NSP1wt control (red). Time points were analysed as two biological replicates of technical triplicates and shown as TCID_50_ ml^−1^. Titres of unpruned and pruned groups were compared via the Mann–Whitney *U* test (*, *P*<0.05).

Following the successful long-term implementation of ouNSP1gfp into SA11 rRVs, the question arose as to whether the segment could tolerate further reductions to the remaining NSP1 ORF region. To that end, we constructed a series of modified ouNSP1gfp segments that systematically truncated the NSP1 ORF sequence directly 5′ of the GFP insert in increments of 9 nt (Fig. 4a). These truncated segments (T1–T8) were next incorporated into the SA11 reverse-genetics system to assess which of them resulted in the rescue of rRVs capable of infecting MA104 cells. After a lengthy incubation of 7–10 days, low levels of CPE and barely detectable GFP expression were observed in cells infected with rRVs containing ouNSP1gfp truncations T1 through T6 (Fig. 4a). No CPE was ever observed from infection attempts of transfections containing T7 and T8 truncations (18 and 9 nt of remaining SA11 5′ ORF sequence, respectively), even after 10 days of observation in six biological replicates. These results suggest that at least 27 nt of the original SA11 ORF must be present for GFP-modified segment recognition and encapsidation into a mature virion, albeit at a much slower rate of replication than either the NSP1wt, NSP1gfp or ouNSP1gfp segments.

Notable differences in performance emerged when the rescued rRVs from the truncated ouNSP1gfp series were subjected to serial passaging. Despite retaining larger SA11 5′ ORF sequences, rRVs containing ouNSP1gfp_T1, T2 or T3 constructs (72, 63 and 54 nt, respectively) were only detectable during passage P1 and failed to produce any CPE or GFP expression even 10 days after P2 infections. In contrast, rRVs containing ouNSP1gfp_T4, T5 or T6 constructs (45, 36 and 27 nt of WT ORF sequence, respectively) were successfully passaged all the way to P10. In electropherotype analyses of these rRVs, severe pruning was observed for the truncated ouNSP1gfp segments, which definitively outcompeted the original modified sequences by passage P10 (Fig. 4b). Closer segment analysis with reverse-transcription PCR showed that pruned variants for T4, T5 and T6 truncations fully replaced the original constructs by passage P10 and were visible as early as passage P5 for the T6 truncation (Fig. 4c). Interestingly, truncated segment pruning was associated with an increase in rRV infectivity, going from barely detectable levels with unpruned stocks (P2 and P3) to a statistically significant rise in infectious virus titres for all three rRVs with pruned segments (P9, P10) (Fig. 4d).

**Fig. 5.**
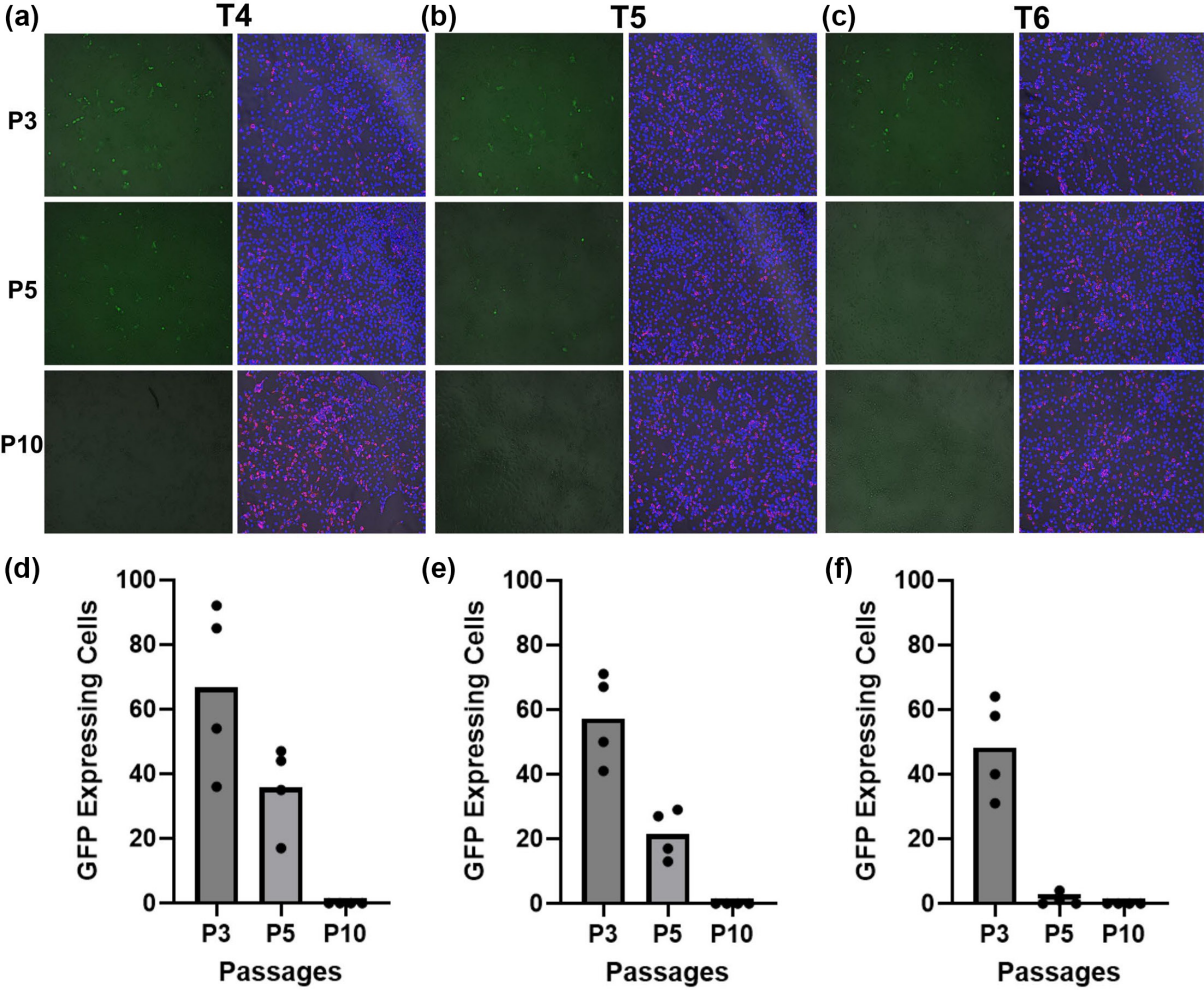
Evaluation of GFP expression in cells infected with truncated ouNSP1gfp rRVs across serial passages. (Left) Fluorescent microscopy images examining brightfield overlayed with GFP expression (green) of live-infected MA104 cells. (Right) Fluorescent microscopy images of methanol-fixed MA104 cells stained to detect host cell nuclei (DAPI, blue) and rotavirus antigen (rabbit anti-SA11 polyclonal sera and AlexaFluor 568 IgG FC, red). Fluorescent microscopy images are provided for cells infected by (**a**) ouNSP1gfp_T4, (**b**) ouNSP1gfp_T5 and (**c**) ouNSP1gfp_T6 rRVs across ten serial passages. Passages P3, P5 and P10 are presented for all rRV lineages. Additional fluorescent images were taken for these samples at predetermined quadrants and used to quantify GFP expression across serial passages for (**d**) ouNSP1gfp_T4 (**e**) ouNSP1gfp_T5 and (**f**) ouNSP1gfp_T6.

Similar to what was observed with larger chimeric NSP1gfp segments, the pruning of truncated ouNSP1gfp segments was associated with diminished functionality for the GFP insert, even as the presence of rotavirus antigen remained consistent across serial passages (Fig. 5a, b and c). rRVs containing truncated ouNSP1gfp sequences presented overall lower GFP expression than their progenitor, which was fully ablated in all cases by passage P10 (Fig. 5c, d and e). Despite being the smallest rescued segment, ouNSP1gfp_T6 presented full ablation of GFP expression by passage P5, suggesting that it was the least stable of the truncated constructs (Fig. 5e). Thus, even though as little as 27 nt of the original NSP1 ORF can be used to incorporate GFP into an rRV, the fitness of the virus continues to favour pruning of the inserted sequence even when the excess length of chimeric segments has been thoroughly mitigated.

### Consistent regions of the GFP insert are pruned across all modified NSP1 segments

**Fig. 6.**
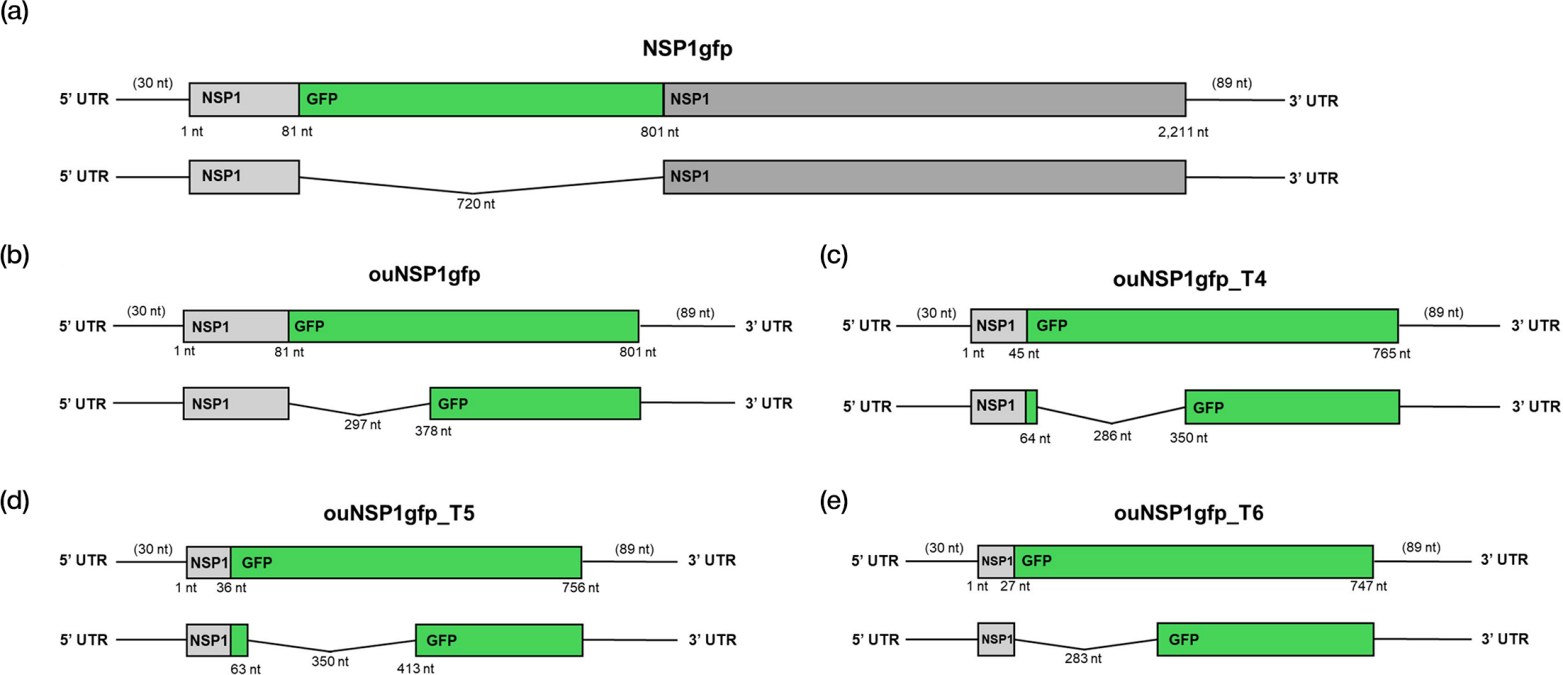
Sequence analysis of pruned NSP1 construct segments across serial passages. Schematics for chimeric segments (**a**) NSP1gfp, (**b**) ouNSP1gfp, (**c**) ouNSP1gfp_T4, (**d**) ouNSP1gfp_T5 and (**e**) ouNSP1gfp_T6 are evaluated at passages P1 (top) and P10 (bottom), based on consensus sequence data obtained from NGS. Schematics follow the same colour-coding established in Fig. 1a. Angled lines denote the size and location of pruned sequences. Numbers denote UTR lengths and nt markers for points of interest along the NSP1 ORF.

Despite a wide variance in sequence size among the chimeric NSP1gfp segments, it was noted that all pruned variants were associated with a pronounced decrease in GFP expression as they overtook the original constructs. This suggests that the sequence pruning was localized to the insert sequence for all constructs. To better assess the excised sequence of the pruned segments, we performed NGS analysis on amplified DNA of the original NSP1gfp, ouNSP1gfp, ouNSP1gfp_T4, ouNSP1gfp_T5 and ouNSP1gfp_T6 segments at passage P1 as well as the pruned counterparts that replaced them at P10 (Fig. 6a-e, full sequences presented in Text S1). Consensus sequence analysis of NSP1gfp segments found that the entirety of the GFP insert was functionally removed by passage P10. Here, the NSP1 ORF regions flanking the insert were not subjected to any sequence pruning, in contrast to the results observed for the P10 analysis of NSP1gfp_BamHI_ (Fig. S2d). In either instance, the minimal pruning of the NSP1 ORF sequence indicates that either the insert sequence is uniquely targeted for pruning or that segments with pruned ORF regions do not confer any selective advantage for rRVs. Population-level analysis of two biological replicates of NSP1gfp rRVs observed the consensus excision of the GFP insert at P10, though multiple haplotypes containing fragments of the insert were still detected at trace levels (Fig. S3). Additionally, NGS analysis of an intermediary NSP1gfp band purified at passage P5 (P5m) observed a consensus sequence with 352 nt pruned from the GFP insert (Fig. S4). These results indicate that the P10 excision of GFP from NSP1gfp segments likely does not occur as a single step, but rather from the continual emergence and selection of progressively pruned subpopulations.

For the P10 consensus sequences of ouNSP1gfp and its T4, T5 and T6 truncations, which lack any NSP1 reading frame downstream of the chimeric insert, sequence excisions between 283 and 350 nt were observed at the 5′ end of the GFP insert. Consensus sequences of P10 ouNSP1gfp and ouNSP1gfp_T6 presented pruning directly at the 5′ terminal sequence of GFP, while the consensus sequences for ouNSP1gfp_T4 and ouNSP1gfp_T5 retained some of the 5′ insert sequence. Moreover, haplotype analysis of the pruned P10 consensus sequence for ouNSP1gfp identified the presence of two additional clones that differ at the 3′ pruning location (Fig. S5). Taken together, these results suggest that the excision patterns of these consensus sequences may be the product of several subpopulations of pruned segments and that the targets of pruning may be less related to sequence motifs of the inserted sequence and possibly more to changes in higher-level structural organization of viral mRNAs imparted by their inclusion.

### Viable truncated and pruned segments retain conserved elements needed for long-range UTR interactions and excise GC-rich regions from inserted sequences

Upon transcription, rotavirus positive-sense (+)RNAs have been documented to form long-range interactions between the 5′ and 3′ terminal sequences [30,32] (Fig. S6a). While the precise functions of these interactions have not been fully determined, it is thought that the resulting conserved secondary structures that emerge from pairing between the 5′ and 3′ UTR sequences produce *cis-*acting elements that are instrumental for the efficient packaging, expression and replication of rotavirus mRNA [30,32]. Indeed, folding analyses of our preliminary candidate NSP1gfp segments demonstrated that these conserved secondary structures were ablated for uuNSP1gfp (Fig. S6b), providing an explanation for why rRVs incorporating this segment were never successfully recovered. To examine the possibility that systematic truncations to the 5′ ORF region of NSP1 resulted in unforeseen consequences to the putative packaging and replication signals, we also conducted folding analyses for the RNAs of ouNSP1gfp modifications T1–T8 (Fig. S6c). Results showed that ouNSP1gfp_T8 (9 nt of NSP1 5′ ORF) exhibited similar destabilized secondary structures to those of uuNSP1gfp, accounting for its failure to produce rRVs. Interestingly, the ouNSP1gfp_T7 construct (never rescued) as well as the T1, T2 and T3 constructs (unrecoverable after P1) exhibited long-range terminal folding interactions similar to constructs that produced viable rRVs. These results suggest that these constructs may have been able to satisfy the mandatory functions required by the long-range terminal sequence interactions and that the ultimate point of failure occurred at a different stage of the rotavirus replication cycle.

**Fig. 7.**
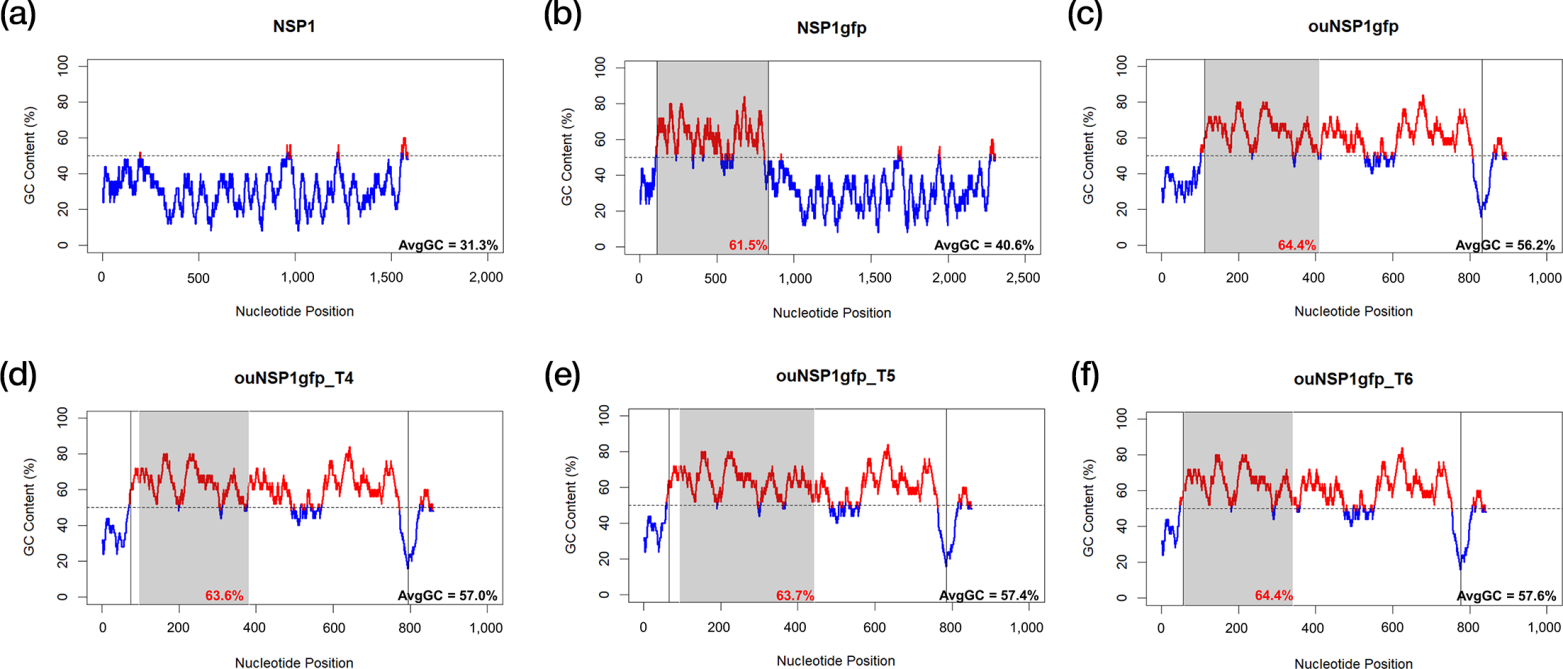
Pruning of chimeric NSP1 segments targets GC-rich insert sequences. (**a**) Visual mapping of the GC content for the WT NSP1 sequence. The average GC percentage of the total sequence – calculated with sliding windows of 25 nt – is displayed in the bottom right corner, while regions of the sequence exceeding a GC threshold of 50% (horizontal dotted line) are indicated in red. GC content mapping is also presented for the chimeric sequences of (**b**) NSP1gfp, (**c**) ouNSP1gfp, (**d**) ouNSP1gfp_T4, (**e**) ouNSP1gfp_T5 and (**f**) ouNSP1gfp under the same measuring constraints. Black vertical lines denote the locations of the inserted GFP sequence, while the grey boxes denote the pruned regions according to respective P10 consensus sequences. The GC percentage of pruned regions is displayed in red text at the bottom right corner of the grey boxes.

We next analysed the P1 and P10 consensus sequences of the chimeric NSP1gfp constructs in order to identify commonalities between the pruned sequences. Despite our previous NGS analyses indicating that the GFP insert region was consistently targeted for pruning, the size and location of each construct’s excised region presented too much variance to suggest that pruning targeted specific sequence motifs. An analysis of secondary structure changes between P1 and P10 constructs highlighted the elimination of hairpin structures within the GFP sequence but were again part of too broad an excised region to determine that their presence was a primary target of sequence pruning (Fig. S7). A broader sequence analysis of WT and chimeric NSP1 segments, however, revealed that the entirety of the GFP insert sequence presents a substantially higher percentage of GC nucleotides (61.5%) than NSP1 (31.3%) (Fig. 7a and b). When P1 and P10 consensus sequences were compared against each other, it was shown that the pruned regions of NSP1gfp, ouNSP1gfp, ouNSP1gfp_T4, ouNSP1gfp_T5 and ouNSP1gfp_T6 excised insert regions corresponded to regions of high-GC concentration (61.5%, 64.4%, 63.6%, 63.7% and 64.4%, respectively) (Fig. 7b–f). These results suggest that insert sequences over a certain GC% threshold, irrespective of segment length, may be a target for segment pruning.

## Discussion

The reverse-genetics system for rotavirus has been successfully leveraged for the incorporation of reporter genes into permissible segments such as NSP1 and NSP3 [5,6, 17,19]. However, chimeric segments with internally inserted sequences have demonstrated long-term instability and are pruned across serial passages [16]. The precise mechanisms for the pruning remain unknown, but the excess length of the chimeric segments was thought to be a driver. Subsequent research has thus developed counter strategies to segment pruning, including the inclusion of a 2A self-cleaving peptide 5′ of the inserted sequence and strategic truncations to the defunct NSP1 ORF sequence 3′ of the insertion [7,16]. Other reports have demonstrated success in generating rRVs that instead utilize a 2A peptide to append the sequence of interest to the 3′ end of the NSP1 and NSP3 segments and have been pursued as a potential foundation for vaccine development [20,23].

This work sought to explore the minimum requirements for the stable, long-term incorporation of chimeric NSP1 segments into rRVs. Our preliminary chimeric NSP1gfp construct was successfully rescued in rRVs but was quickly outcompeted by a pruned variant across serial passaging, recapitulating previous findings [16]. However, we found that the entire NSP1 ORF sequence 3′ of the GFP insert could be deleted with no consequence (ouNSP1gfp), resulting in rRVs that presented enhanced segment stability and GFP expression up to passage P10. Through systematic truncations to the ORF sequence 5′ of the GFP insert, we further demonstrated that only 27 nt of the original NSP1 reading frame is necessary for the rescue (ouNSP1gfp_T6). Notably, conserved secondary structures arising from long-range UTR interactions necessary for rotavirus gene expression and replication are only disrupted in constructs presenting ≤9 nt of the NSP1 ORF (uuNSP1gfp, ouNSP1gfp_T8), suggesting that even smaller constructs may be achievable. We additionally showed that reverse-genetics transfections, absent any NSP1 segment (ΔNSP1), failed to recover rRVs. These results could be interpreted to mean that recognition of all 11 segments is required for the formation of mature rotavirus particles, regardless of intact function. Structural studies have observed that the dsRNA segments present a semi-condensed, concentric organization within the rotavirus capsid [33], which has been hypothesized to facilitate individualized interactions with the internal VP1/VP3 transcription complexes [34]. While the capsid theoretically provides independent transcription complexes for up to 12 segments, it is possible that 10 segments may not provide sufficient structural organization necessary to promote efficient transcription [33]. Alternatively, the missing segment may result in points of failure elsewhere in the rotavirus lifecycle, as it has been theorized that interactions with all 11+RNAs are critical for encapsidation and virus particle assembly in the virioplasm [35,36].

Despite successful incorporation, it appears that these truncation modifications to NSP1gfp merely delay, rather than avoid, segment pruning. Though ouNSP1gfp rRVs continued to express GFP by passage P10, reverse-transcription PCR analysis of their respective DNA amplicons revealed the growing presence of a pruned variant that was not visible on the electropherotype gel. We interpret these results to indicate that, despite the reduced initial size relative to the NSP1wt segment, ouNSP1gfp still produces subpopulations of pruned segments that would eventually outcompete the original construct given enough serial passages. Pruned segments were also observed for the even smaller T4, T5 and T6 variants of ouNSP1gfp to a more dramatic effect. Not only were these segments fully replaced by insert-pruned variants by passage P10, but the gradual succession of these pruned variants was correlated with an increase in rRV titres in passages P9 and P10.

Several factors appear to be at play regarding the emergence and replacement of pruned chimeric segments in rRVs. First, it has been demonstrated that the replication efficiency of rotavirus mRNAs is inversely impacted by template size [37]. This provides a rationale for why rRVs preferentially select for the pruned variant, even in cases where the original NSP1gfp construct was substantially smaller than the WT segment. Also of interest is that the insert sequence for all versions of the NSP1gfp constructs was targeted for pruning, as the non-sequence-specific manner of the excision suggests a general incompatibility with the insert itself. The +RNA sequences for all 11 rotavirus segments are notable for their high AU content [37], averaging 66.2% among SA11 segments (Fig. S8). As the AU percentage is also correlated with replication efficiency [37], a possible explanation is thus provided for the region-specific pruning of the GC-rich GFP insert that is preferentially selected over the original construct. A potential explanation is additionally provided for why rRVs containing T4, T5 or T6 modifications of ouNSP1gfp – near total replacements of the NSP1 ORF sequence with GFP – exhibited poor infectivity until the emergence and selection of their respective pruned sequences. However, we note that pruned versions of all chimeric segments, despite increased performance over their unpruned counterparts, display delayed replication and reduced endpoint titres compared to wild-type controls (Figs. 2 and 4). As these truncated segments are definitionally incapable of restoring the original functions of the NSP1 segment through insert pruning, the possibility arises that pruned variants may still be impacted by their diminished ability to suppress the host cell interferon response.

Further research is required to interrogate how chimeric rotavirus segments or their pruned counterparts are preferentially incorporated into rRVs. The results of this study strongly suggest that GC-rich rotavirus+RNAs result in subpopulations of pruned segments whose improved replication efficiency eventually outpaces the original construct. Moreover, the reverse-genetics system may be leveraged to better quantify the differential integration of NSP1 segments of various sizes and insert sequence content. Additional research is also needed to explore the failure to pass rRVs containing T1, T2 and T3 modifications for ouNSP1gfp beyond a single passage, as well as the failure to rescue rRVs containing ouNSP1gfp_T7. Predictive folding analyses indicated that the +RNAs of these constructs retained critical translation and replication enhancers formed by long-range UTR interactions. The presence of these elements is perhaps a necessary but not sufficient condition for viable rRVs, and given the documented non-viability of ΔNSP1 transfections, it is possible that these constructs exhibited a replicative efficiency too poor to sustain an rRV population before pruned alternatives could emerge. The substitution of GFP with different reporter genes is one potential way to test this hypothesis. To that end, we note the specific mechanisms of sequence pruning remain poorly understood, as well as whether it can be circumvented by inserting sequences below a certain GC% threshold. Clinical reports in the literature have documented the existence of human RVs with AU-rich insertions of up to 148 nt in the 3′ UTR of NSP5/6 [38], suggesting the tolerance for certain types of sequences. Therefore, the application of these chimeric segment strategies to lower GC sequences of interest may present a valuable future direction for the development of multi-virus vaccines.

## Supplementary material

10.1099/jgv.0.002275 Supplementary Material 1.

## Abbreviations

CPEs: cytopathic effects
EMEM: Eagle’s Minimum Essential Medium
GFP: Green Fluorescent Protein
G-MEM: Glasgow’s Minimal Essential Medium
HPI: Hours post-infection
NGS: next-generation sequencing
ORF: Open reading frame
rRV: recombinant rotavirus
RVs: rotaviruses
UTR: Untranslated region
WT: Wild type
