# Supplementary material for: Probing the sequence constraints for the stable incorporation of chimeric NSP1 segments into infectious rotaviruses
Source: J Gen Virol. 2026 Jul 3;107(7):002275. doi: 10.1099/jgv.0.002275 (PMC13338669; doi:10.1099/jgv.0.002275)
Supplement: Supplementary Material 1. [file jgv-107-02275-s001.pdf]

Supplementary Information

Title: **Probing the sequence constraints for the stable incorporation of chimeric NSP1 segments into infectious rotaviruses**

Authors: **Joseph Kendra, Gabriel I. Parra**

Affiliation: **Division of Viral Products, Center for Biologics Evaluation and Research, Food and Drug Administration, Silver Spring, Maryland, United States of America**

pT7 NSP1 Insert

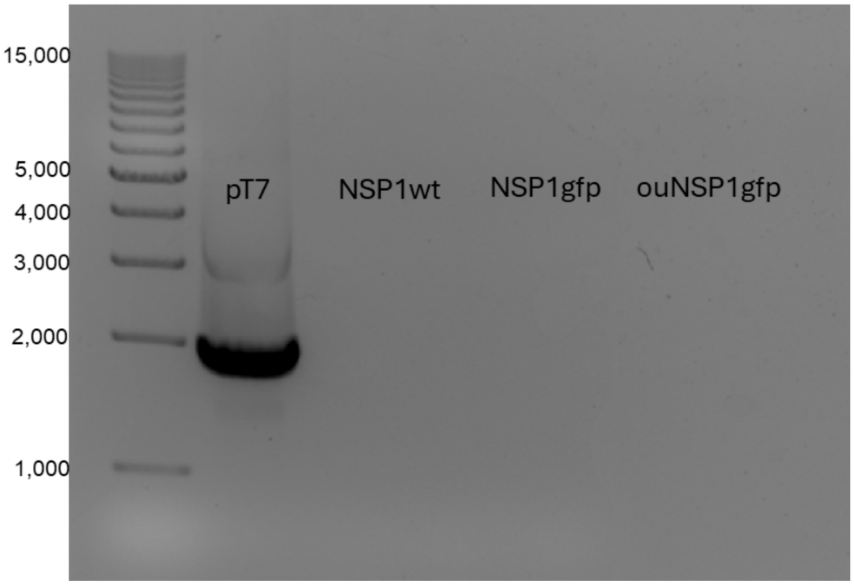

Figure S1. Validation of rescued rRVs containing chimeric NSP1 segments

The presence of lingering pT7 reverse genetics plasmids in rescued rRV stocks was investigated via PCR using primers specific to the plasmid backbone. Negative PCR results for WT, NSP1gfp, and ouNSP1gfp rRVs are shown next to a pT7-NSP1SA11 control.

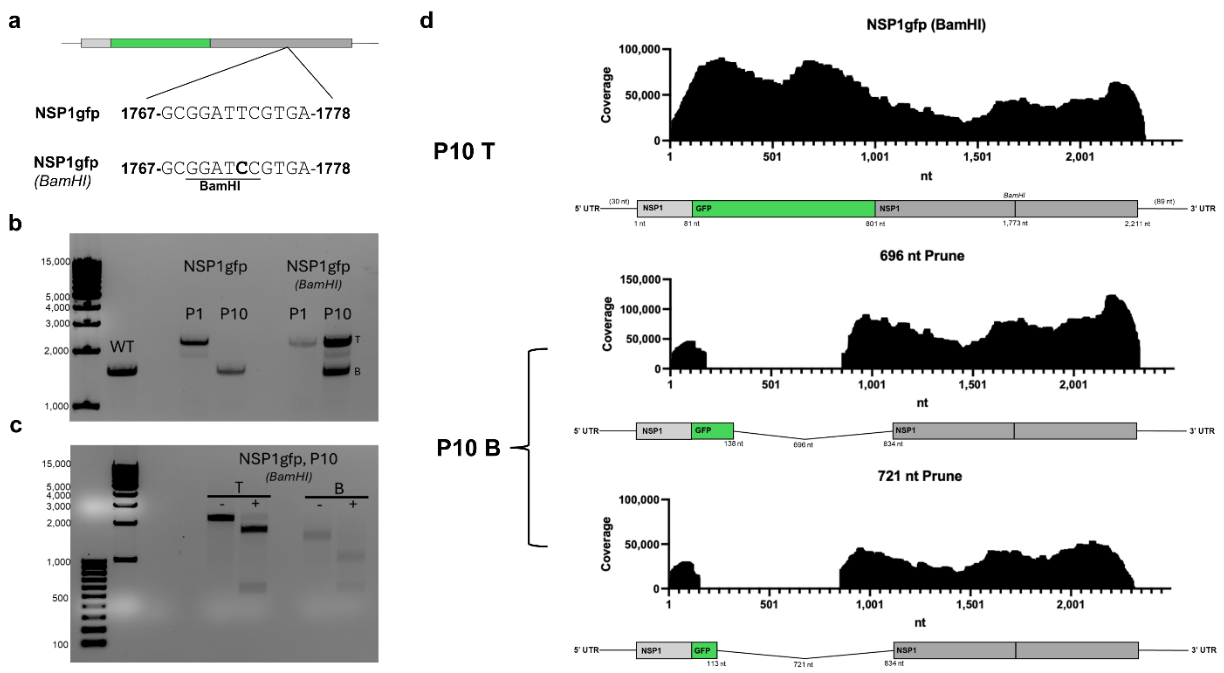

Figure S2.

(a) Schematic of NSP1gfp segment indicating the region where a silent mutation was introduced to develop NSP1gfp$_{BamHI}$. The mutation is indicated in bold and the resulting restriction site is underlined. **(b)** Agarose gel of reverse transcription PCR cDNA products for NSP1wt, as well as NSP1gfp and NSP1gfp$_{BamHI}$ at passages P1 and P10. An unpruned (T) and pruned (B) segment was observed for NSP1gfp$_{BamHI}$ at passage P10, which were harvested for restriction enzyme digest analysis. **(c)** Purified T and B bands from NSP1gfp$_{BamHI}$ P10 in the presence (+) or absence (-) of BamHI restriction digestion. **(d)** Sequence read coverage of NGS analysis for NSP1gfp$_{BamHI}$ P10 T, as well as two pruned subpopulations identified for P10 B. The impact of pruning on the NSP1gfp$_{BamHI}$ sequence is represented with accompanying segment schematics.

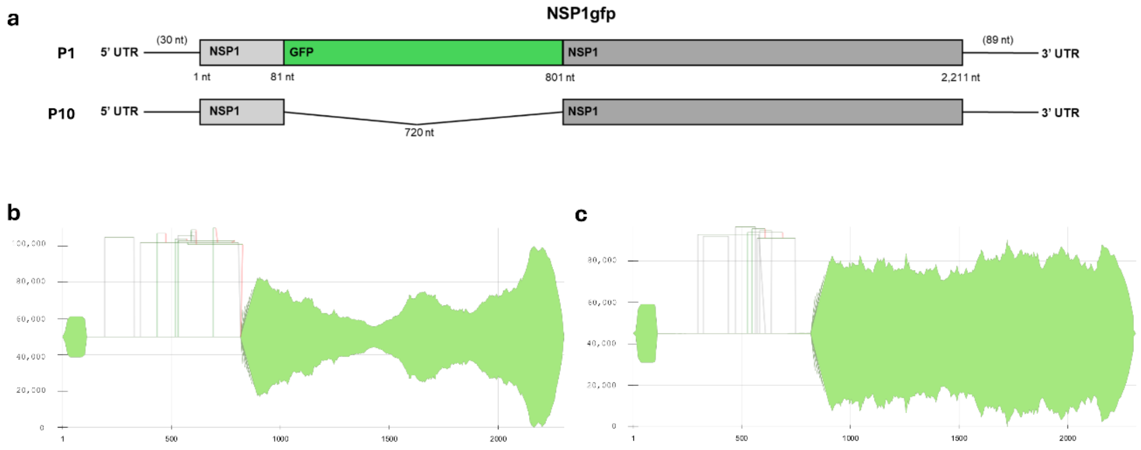

Figure S3. Virus populations carrying differentially pruned NSP1gfp segments are detectable at passage P10

(a) Schematic of NSP1gfp segment consensus sequences at passages P1 and P10, depicting the full excision of the GFP insert sequence from rRVs by the end of serial passaging. Population analyses of two biological replicates of P10 NSP1gfp **(b** and **c)** in which consensus read coverage is mapped to the NSP1gfp reference sequence. Haplotype reconstruction was used to identify alternative clones, denoted as parallel lines within areas of low coverage and are indicative of instances of non-uniform pruning within the population.

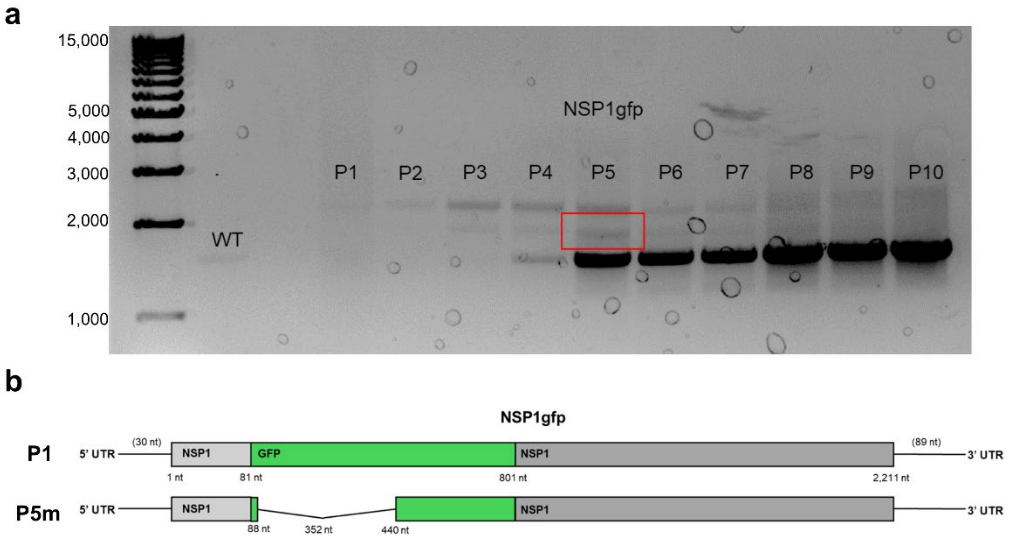

Figure S4. Identification of intermediate pruned NSP1gfp segment in serial passage P5

(a) Agarose gel of NSP1 segment cDNA amplified from WT NSP1 and serial passages of NSP1gfp. Between the bands corresponding to full length and fully pruned NSP1gfp segments, a middle band is faintly observed between passages P3 to P8. A red box highlights the middle band from passage P5 (P5m) that was taken for NGS analysis. **(b)** Schematics of the NSP1gfp sequence at P1 compared to the consensus sequence obtained at P5m. Angled lines denote the size and location of the pruned region.

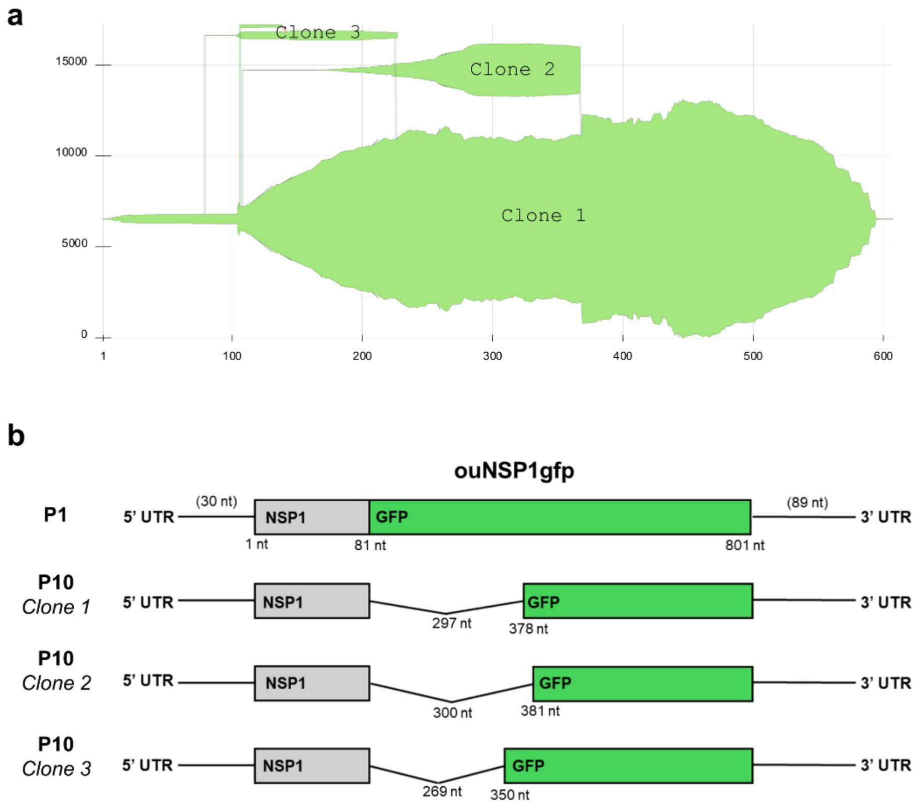

Figure S5. Identification of alternatively pruned subpopulations for P10 ouNSP1gfp

(a) Haplotype analysis of the P10 sequences of ouNSP1gfp obtained through NGS analysis. The pruned sequence for P10 ouNSP1gfp identified in Fig 5B is used as a reference sequence, and read coverage is graphically represented as Sankey diagrams. The majority of reads (Clone 1) map to the pruned consensus sequence, but parallel lines stemming from bifurcations (Clone 2, Clone 3) denote the presence of virus subpopulations with pruning variance on the 3' side. **(b)** Schematics of the respective pruning for clones 1,2, and 3 of P10 ouNSP1gfp, respectively. Angled lines denote the size and location of the pruned region.

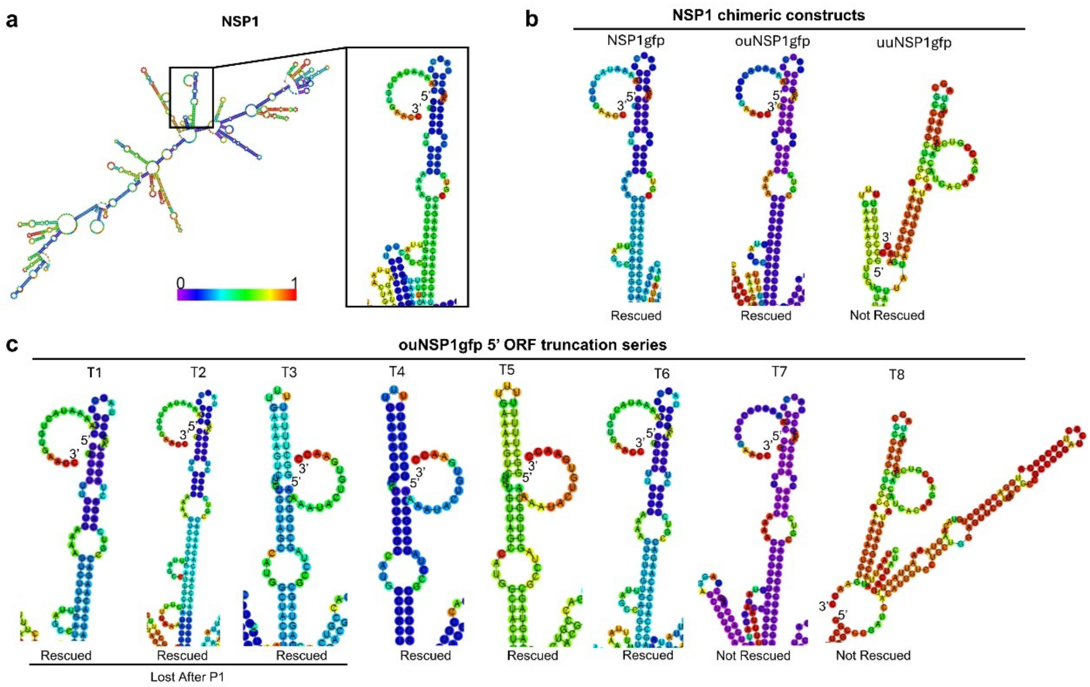

Figure 6. Evaluation of long-range terminal sequence interactions of NSP1gfp constructs

(a) *(Left)* Minimum free energy model for the secondary structure of the WT NSP1 +RNA. Nucleotide colors denote base-pair probability, in which colors closer to 1 (red) indicate a higher probability of being unpaired. The conserved structures formed by long-range interactions with the 5' and 3' terminal sequences are highlighted with a black box. *(Right)* A zoomed-in image of the terminal sequence interaction, with the respective 5' and 3' ends labeled. **(b)** Predicted long-range terminal sequence interactions for NSP1 chimeric segments NSP1gfp, ouNSP1gfp, and uuNSP1gfp. Structures are labeled with the 5' and 3' ends of the sequence, as well as whether rRVs transfected with these constructs were successfully rescued. **(c)** Predicted secondary structures of the terminal sequence interactions for truncated ouNSP1gfp constructs T1 through T8. Structures are labeled with the 5' and 3' ends of the sequence, as well as whether rRVs transfected with these constructs were successfully rescued. For constructs T1 through T3, it is additionally noted that rescued rRVs were lost after a single round of passaging.

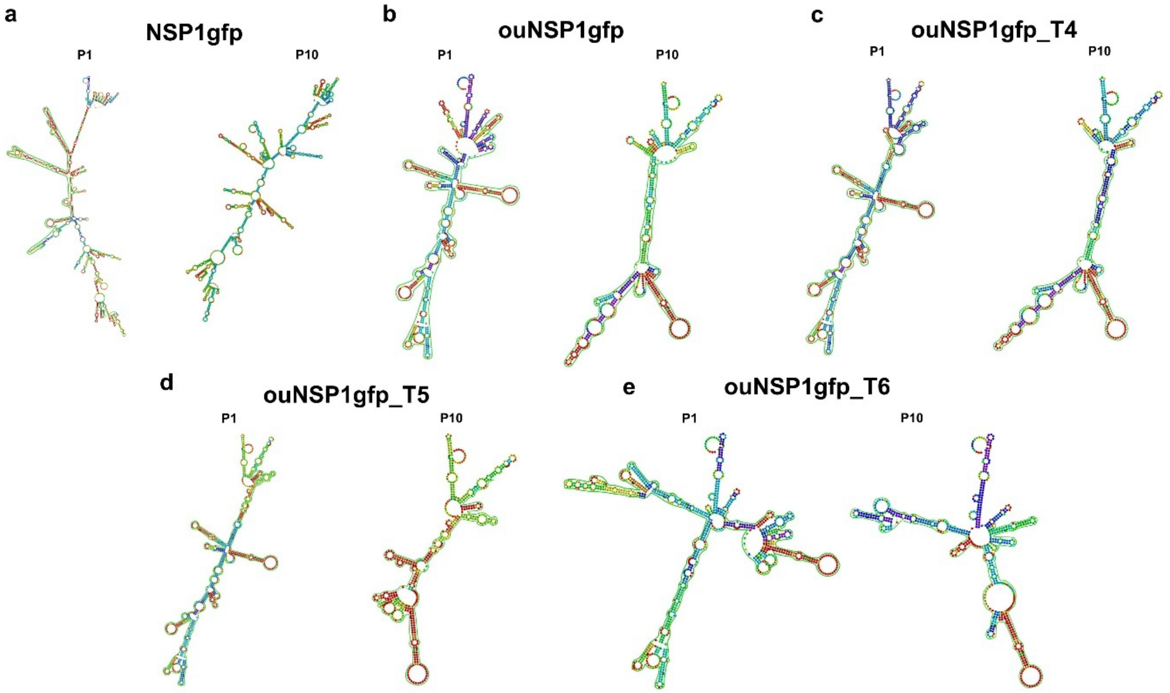

Figure S7. Predicted secondary structures of chimeric NSP1 +RNAs and pruned variants

Minimum free energy models for the predicted secondary structures of chimeric NSP1 constructs that allowed for the rescue and serial passaging of infectious rRVs: **(a)** NSP1gfp, **(b)** ouNSP1gfp, **(c)** ouNSP1gfp_T4, **(d)** ouNSP1gfp_T5, **(e)** ouNSP1gfp_T6. Nucleotide colors denote base pair probability, in which warmer colors (e.g. red) indicate a higher probability of being unpair. “P1” structures refer to the predicted +RNA structures for the original constructs, while “P10” refer to the +RNA of the pruned variant segments that were isolated at the end of serial passaging. Green outlines around the secondary structures denote the inserted GFP sequence.

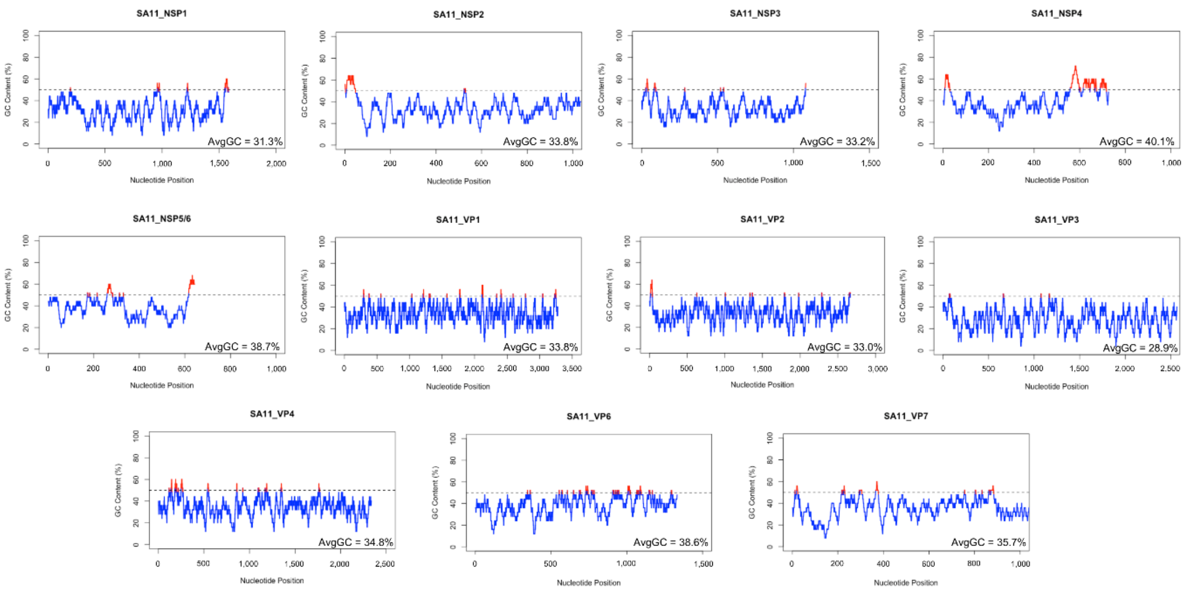

Figure S8. Genomic segments for SA11 rotavirus all present highly AU rich sequences

Visual mapping of the GC content for the genomic segments of SA11 rotavirus. The average GC percentage for each sequence is displayed in the bottom right corner, while regions of the sequence exceeding a GC threshold of 50% (horizontal dotted line) are indicated in red.

Supplemental Text

Original construct sequences for SA11 NSP1

Inserted GFP sequence (MN968806) is represented with green highlights. The locations of point mutations are highlighted in red. Excised regions of pruned sequences are represented with a “^” character.

>SA11 NSP1 (LC178570.1)

GGCTTTTTTTTGAAAAGTCTTGTGTTAGCCATGGCTACTTTTAAAGATGCATGCTTTCATTATCGTAGAT
TAACTGCTTTAAATCGGAGATTATGCAACATTGGTGCAAATTCTATTTGGATGCCAGTTCCTGATGCGAA
AATTAAGGGGTGGTGTTTAGAATGTTGTCAAATAGCTGATTTAACCCATTGTTATGGTTGCTCATTGCCG
CATGTTTGCAAATGGTGTGTTCAGAACAGAAGATGCTTCCTTGACAATGAACCTCATTTGCTTAAGCTTA
GAACTGTGAAACATCCAATTACCAAAGACAAATTACAGTGTATCATAGACTTGTACAATATAATATTTCC
AATTAATGATAAAGTAATTAGAAAATTTGAAAGAATGATAAAGCAAAGAGAATGTAGGAATCAATATAAA
ATTGAATGGTATAATCATTTGCTGCTCCCAATTACATTAAATGCTGCTGCATTTAAGTTTGATGAAAATA
ATCTTTATTATGTTTTTGGGTTATATGAGAAATCAGTCAGTGATATATATGCTCCATATAGAATTGTTAA
CTTTATAAATGAATTTGATAAATTATTGCTTGATCATATTAACTTTACAAGAATGTCCAATCTACCAATA
GAGTTGAGAAACCATTACGCAAAGAAATACTTCCAATTATCAAGACTGCCATCATCAAAACTAAAGCAAA
TTTACTTTTCAGATTTTACTAAAGAAACTGTGATTTTTAATACTTATACAAAAACGCCAGGAAGATCAAT
ATACAGAAATGTAACTGAATTTAATTGGAGAGATGAATTGGAGCTTTATTCTGATTTAAAAAATGATAAG
AATAAATTAATTGCTGCAATGATGACGAGTAAGTATACTCGGTTCTATGCTCATGATAATAATTTTGGAA
GGTTGAAAATGACAATATTTGAGTTGGGACATCATTGTCAGCCTAACTACGTGGCATCTAATCACCCAGG
CAATGCTTCCGATATCCAGTACTGTAAATGGTGTAATATAAAATATTTTCTTAGTAAAATTGATTGGCGG
ATTCGTGATATGTATAATTTATTGATGGAATTTATTAAGGATTGTTATAAAAGTAATGTTAACGTTGGAC
ATTGTAGTTCTGTTGAAAACATATATCCTTTAATTAAAAGATTAATTTGGAGTTTGTTTACTAATCACAT
GGTCAAACAATTGAAGAAGTGTTTAATCACATGTCGCCAGTGTCAGTTGAAGGTACGAATGTCATCATGT
TGATTCTTGGATTGAATATTAGTTTGTATAATGAAATTAAGCGCACTTTGAATGTAGATAGCATACCAAT
GGTACTTAATTTAAATGAATTCAGTAGTATAGTTAAATCAATTAGCAGTAAATGGTATAATGTTGATGAA
TTGGATAAATTGCCAATGTCAATAAAATCAACGGAGGAACTGATTGAAATGAAGAATTCTGGAACTTTAA
CTGAAGAATTTGAGCTACTGATCTCCAACTCAGAAGATGACAATGAGTGAAATTATGTCACTATCTAATT
ATACAGTATTTAGCCATCACAAGACCGTCCAGACTAGAGTAGCGCCTAGCTGGCAAAATACTGTGAACC

>SA11_NSP1gfp

GGCTTTTTTTTGAAAAGTCTTGTGTTAGCCATGGCTACTTTTAAAGATGCATGCTTTCATTATCGTAGAT
TAACTGCTTTAAATCGGAGATTATGCAACATTGGTGCAAATATGGTGAGCAAGGGCGAGGAGCTGTTCAC
CGGGGTGGTGCCCATCCTGGTCGAGCTGGACGGCGACGTAAACGGCCACAAGTTCAGCGTGTCCGGCGAG
GGCGAGGGCGATGCCACCTACGGCAAGCTGACCCTGAAGTTCATCTGCACCACCGGCAAGCTGCCCGTGC
CCTGGCCCACCCTCGTGACCACCCTGACCTACGGCGTGCAGTGCTTCAGCCGCTACCCCGACCACATGAA
GCAGCACGACTTCTTCAAGTCCGCCATGCCCGAAGGCTACGTCCAGGAGCGCACCATCTTCTTCAAGGAC
GACGGCAACTACAAGACCCGCGCCGAGGTGAAGTTCGAGGGCGACACCCTGGTGAACCGCATCGAGCTGA
AGGGCATCGACTTCAAGGAGGACGGCAACATCCTGGGGCACAAGCTGGAGTACAACTACAACAGCCACAA
CGTCTATATCATGGCCGACAAGCAGAAGAACGGCATCAAGGTGAACTTCAAGATCCGCCACAACATCGAG
GACGGCAGCGTGCAGCTCGCCGACCACTACCAGCAGAACACCCCCATCGGCGACGGCCCCGTGCTGCTGC
CCGACAACCACTACCTGAGCACCCAGTCCGCCCTGAGCAAAGACCCCAACGAGAAGCGCGATCACATGGT
CCTGCTGGAGTTCGTGACCGCCGCCGGGATCACTCTCGGCATGGACGAGCTGTACAAGTAATCTATTTGG
ATGCCAGTTCCTGATGCGAAAATTAAGGGGTGGTGTTTAGAATGTTGTCAAATAGCTGATTTAACCCATT
GTTATGGTTGCTCATTGCCGCATGTTTGCAAATGGTGTGTTCAGAACAGAAGATGCTTCCTTGACAATGA
ACCTCATTTGCTTAAGCTTAGAACTGTGAAACATCCAATTACCAAAGACAAATTACAGTGTATCATAGAC
TTGTACAATATAATATTTCCAATTAATGATAAAGTAATTAGAAAATTTGAAAGAATGATAAAGCAAAGAG
AATGTAGGAATCAATATAAAATTGAATGGTATAATCATTTGCTGCTCCCAATTACATTAAATGCTGCTGC
ATTTAAGTTTGATGAAAATAATCTTTATTATGTTTTTGGGTTATATGAGAAATCAGTCAGTGATATATAT

GCTCCATATAGAATTGTTAACTTTATAAATGAATTTGATAAATTATTGCTTGATCATATTAACTTTACAA
GAATGTCCAATCTACCAATAGAGTTGAGAAACCATTACGCAAAGAAATACTTCCAATTATCAAGACTGCC
ATCATCAAAACTAAAGCAAATTTACTTTTCAGATTTTACTAAAGAAACTGTGATTTTTAATACTTATACA
AAAACGCCAGGAAGATCAATATACAGAAATGTAACTGAATTTAATTGGAGAGATGAATTGGAGCTTTATT
CTGATTTAAAAAATGATAAGAATAAATTAATTGCTGCAATGATGACGAGTAAGTATACTCGGTTCTATGC
TCATGATAATAATTTTGGAAGGTTGAAAATGACAATATTTGAGTTGGGACATCATTGTCAGCCTAACTAC
GTGGCATCTAATCACCCAGGCAATGCTTCCGATATCCAGTACTGTAAATGGTGTAATATAAAATATTTTC
TTAGTAAAATTGATTGGCGGATTCGTGATATGTATAATTTATTGATGGAATTTATTAAGGATTGTTATAA
AAGTAATGTTAACGTTGGACATTGTAGTTCTGTTGAAAACATATATCCTTTAATTAAAAGATTAATTTGG
AGTTTGTTTACTAATCACATGGATCAAACAATTGAAGAAGTGTTTAATCACATGTCGCCAGTGTCAGTTG
AAGGTACGAATGTCATCATGTTGATTCTTGGATTGAATATTAGTTTGTATAATGAAATTAAGCGCACTTT
GAATGTAGATAGCATACCAATGGTACTTAATTTAAATGAATTCAGTAGTATAGTTAAATCAATTAGCAGT
AAATGGTATAATGTTGATGAATTGGATAAATTGCCAATGTCAATAAAATCAACGGAGGAACTGATTGAAA
TGAAGAATTCTGGAACTTTAACTGAAGAATTTGAGCTACTGATCTCCAACTCAGAAGATGACAATGAGTG
AAATTATGTCACTATCTAATTATACAGTATTTAGCCATCACAAGACCGTCCAGACTAGAGTAGCGCCTAG
CTGGCAAAATACTGTGAACC

>SA11_NSP1gfp_BamHI

GGCTTTTTTTTGAAAAGTCTTGTGTTAGCCATGGCTACTTTTAAAGATGCATGCTTTCATTATCGTAGAT
TAACTGCTTTAAATCGGAGATTATGCAACATTGGTGCAAATATGGTGAGCAAGGGCGAGGAGCTGTTCAC
CGGGGTGGTGCCCATCCTGGTCGAGCTGGACGGCGACGTAAACGGCCACAAGTTCAGCGTGTCCGGCGAG
GGCGAGGGCGATGCCACCTACGGCAAGCTGACCCTGAAGTTCATCTGCACCACCGGCAAGCTGCCCGTGC
CCTGGCCCACCCTCGTGACCACCCTGACCTACGGCGTGCAGTGCTTCAGCCGCTACCCCGACCACATGAA
GCAGCACGACTTCTTCAAGTCCGCCATGCCCGAAGGCTACGTCCAGGAGCGCACCATCTTCTTCAAGGAC
GACGGCAACTACAAGACCCGCGCCGAGGTGAAGTTCGAGGGCGACACCCTGGTGAACCGCATCGAGCTGA
AGGGCATCGACTTCAAGGAGGACGGCAACATCCTGGGGCACAAGCTGGAGTACAACTACAACAGCCACAA
CGTCTATATCATGGCCGACAAGCAGAAGAACGGCATCAAGGTGAACTTCAAGATCCGCCACAACATCGAG
GACGGCAGCGTGCAGCTCGCCGACCACTACCAGCAGAACACCCCCATCGGCGACGGCCCCGTGCTGCTGC
CCGACAACCACTACCTGAGCACCCAGTCCGCCCTGAGCAAAGACCCCAACGAGAAGCGCGATCACATGGT
CCTGCTGGAGTTCGTGACCGCCGCCGGGATCACTCTCGGCATGGACGAGCTGTACAAGTAATCTATTTGG
ATGCCAGTTCCTGATGCGAAAATTAAGGGGTGGTGTTTAGAATGTTGTCAAATAGCTGATTTAACCCATT
GTTATGGTTGCTCATTGCCGCATGTTTGCAAATGGTGTGTTCAGAACAGAAGATGCTTCCTTGACAATGA
ACCTCATTTGCTTAAGCTTAGAACTGTGAAACATCCAATTACCAAAGACAAATTACAGTGTATCATAGAC
TTGTACAATATAATATTTCCAATTAATGATAAAGTAATTAGAAAATTTGAAAGAATGATAAAGCAAAGAG
AATGTAGGAATCAATATAAAATTGAATGGTATAATCATTTGCTGCTCCCAATTACATTAAATGCTGCTGC
ATTTAAGTTTGATGAAAATAATCTTTATTATGTTTTTGGGTTATATGAGAAATCAGTCAGTGATATATAT
GCTCCATATAGAATTGTTAACTTTATAAATGAATTTGATAAATTATTGCTTGATCATATTAACTTTACAA
GAATGTCCAATCTACCAATAGAGTTGAGAAACCATTACGCAAAGAAATACTTCCAATTATCAAGACTGCC
ATCATCAAAACTAAAGCAAATTTACTTTTCAGATTTTACTAAAGAAACTGTGATTTTTAATACTTATACA
AAAACGCCAGGAAGATCAATATACAGAAATGTAACTGAATTTAATTGGAGAGATGAATTGGAGCTTTATT
CTGATTTAAAAAATGATAAGAATAAATTAATTGCTGCAATGATGACGAGTAAGTATACTCGGTTCTATGC
TCATGATAATAATTTTGGAAGGTTGAAAATGACAATATTTGAGTTGGGACATCATTGTCAGCCTAACTAC
GTGGCATCTAATCACCCAGGCAATGCTTCCGATATCCAGTACTGTAAATGGTGTAATATAAAATATTTTC
TTAGTAAAATTGATTGGCGGAT**C**CGTGATATGTATAATTTATTGATGGAATTTATTAAGGATTGTTATAA
AAGTAATGTTAACGTTGGACATTGTAGTTCTGTTGAAAACATATATCCTTTAATTAAAAGATTAATTTGG
AGTTTGTTTACTAATCACATGGATCAAACAATTGAAGAAGTGTTTAATCACATGTCGCCAGTGTCAGTTG
AAGGTACGAATGTCATCATGTTGATTCTTGGATTGAATATTAGTTTGTATAATGAAATTAAGCGCACTTT
GAATGTAGATAGCATACCAATGGTACTTAATTTAAATGAATTCAGTAGTATAGTTAAATCAATTAGCAGT
AAATGGTATAATGTTGATGAATTGGATAAATTGCCAATGTCAATAAAATCAACGGAGGAACTGATTGAAA
TGAAGAATTCTGGAACTTTAACTGAAGAATTTGAGCTACTGATCTCCAACTCAGAAGATGACAATGAGTG

AAATTATGTCACTATCTAATTATACAGTATTTAGCCATCACAAGACCGTCCAGACTAGAGTAGCGCCTAG
CTGGCAAAATACTGTGAACC

>SA11_ouNSP1gfp

GGCTTTTTTTTGAAAAGTCTTGTGTTAGCCATGGCTACTTTTAAAGATGCATGCTTTCATTATCGTAGAT
TAACTGCTTTAAATCGGAGATTATGCAACATTGGTGCAAATATGGTGAGCAAGGGCGAGGAGCTGTTCAC
CGGGGTGGTGCCCATCCTGGTCGAGCTGGACGGCGACGTAAACGGCCACAAGTTCAGCGTGTCCGGCGAG
GGCGAGGGCGATGCCACCTACGGCAAGCTGACCCTGAAGTTCATCTGCACCACCGGCAAGCTGCCCGTGC
CCTGGCCCACCCTCGTGACCACCCTGACCTACGGCGTGCAGTGCTTCAGCCGCTACCCCGACCACATGAA
GCAGCACGACTTCTTCAAGTCCGCCATGCCCGAAGGCTACGTCCAGGAGCGCACCATCTTCTTCAAGGAC
GACGGCAACTACAAGACCCGCGCCGAGGTGAAGTTCGAGGGCGACACCCTGGTGAACCGCATCGAGCTGA
AGGGCATCGACTTCAAGGAGGACGGCAACATCCTGGGGCACAAGCTGGAGTACAACTACAACAGCCACAA
CGTCTATATCATGGCCGACAAGCAGAAGAACGGCATCAAGGTGAACTTCAAGATCCGCCACAACATCGAG
GACGGCAGCGTGCAGCTCGCCGACCACTACCAGCAGAACACCCCCATCGGCGACGGCCCCGTGCTGCTGC
CCGACAACCACTACCTGAGCACCCAGTCCGCCCTGAGCAAAGACCCCAACGAGAAGCGCGATCACATGGT
CCTGCTGGAGTTCGTGACCGCCGCCGGGATCACTCTCGGCATGGACGAGCTGTACAAGTAAAATTATGTC
ACTATCTAATTATACAGTATTTAGCCATCACAAGACCGTCCAGACTAGAGTAGCGCCTAGCTGGCAAAAT
ACTGTGAACC

>SA11_ouNSP1gfp_T4

GGCTTTTTTTTGAAAAGTCTTGTGTTAGCCATGGCTACTTTTAAAGATGCATGCTTTCATTATCGTAGAT
TAACTATGGTGAGCAAGGGCGAGGAGCTGTTCACCGGGGTGGTGCCCATCCTGGTCGAGCTGGACGGCGA
CGTAAACGGCCACAAGTTCAGCGTGTCCGGCGAGGGCGAGGGCGATGCCACCTACGGCAAGCTGACCCTG
AAGTTCATCTGCACCACCGGCAAGCTGCCCGTGCCCTGGCCCACCCTCGTGACCACCCTGACCTACGGCG
TGCAGTGCTTCAGCCGCTACCCCGACCACATGAAGCAGCACGACTTCTTCAAGTCCGCCATGCCCGAAGG
CTACGTCCAGGAGCGCACCATCTTCTTCAAGGACGACGGCAACTACAAGACCCGCGCCGAGGTGAAGTTC
GAGGGCGACACCCTGGTGAACCGCATCGAGCTGAAGGGCATCGACTTCAAGGAGGACGGCAACATCCTGG
GGCACAAGCTGGAGTACAACTACAACAGCCACAACGTCTATATCATGGCCGACAAGCAGAAGAACGGCAT
CAAGGTGAACTTCAAGATCCGCCACAACATCGAGGACGGCAGCGTGCAGCTCGCCGACCACTACCAGCAG
AACACCCCCATCGGCGACGGCCCCGTGCTGCTGCCCGACAACCACTACCTGAGCACCCAGTCCGCCCTGA
GCAAAGACCCCAACGAGAAGCGCGATCACATGGTCCTGCTGGAGTTCGTGACCGCCGCCGGGATCACTCT
CGGCATGGACGAGCTGTACAAGTAAAATTATGTCACTATCTAATTATACAGTATTTAGCCATCACAAGAC
CGTCCAGACTAGAGTAGCGCCTAGCTGGCAAAATACTGTGAACC

>SA11_ouNSP1gfp_T5

GGCTTTTTTTTGAAAAGTCTTGTGTTAGCCATGGCTACTTTTAAAGATGCATGCTTTCATTATCGTATGG
TGAGCAAGGGCGAGGAGCTGTTCACCGGGGTGGTGCCCATCCTGGTCGAGCTGGACGGCGACGTAAACGG
CCACAAGTTCAGCGTGTCCGGCGAGGGCGAGGGCGATGCCACCTACGGCAAGCTGACCCTGAAGTTCATC
TGCACCACCGGCAAGCTGCCCGTGCCCTGGCCCACCCTCGTGACCACCCTGACCTACGGCGTGCAGTGCT
TCAGCCGCTACCCCGACCACATGAAGCAGCACGACTTCTTCAAGTCCGCCATGCCCGAAGGCTACGTCCA
GGAGCGCACCATCTTCTTCAAGGACGACGGCAACTACAAGACCCGCGCCGAGGTGAAGTTCGAGGGCGAC
ACCCTGGTGAACCGCATCGAGCTGAAGGGCATCGACTTCAAGGAGGACGGCAACATCCTGGGGCACAAGC
TGGAGTACAACTACAACAGCCACAACGTCTATATCATGGCCGACAAGCAGAAGAACGGCATCAAGGTGAA
CTTCAAGATCCGCCACAACATCGAGGACGGCAGCGTGCAGCTCGCCGACCACTACCAGCAGAACACCCCC
ATCGGCGACGGCCCCGTGCTGCTGCCCGACAACCACTACCTGAGCACCCAGTCCGCCCTGAGCAAAGACC
CCAACGAGAAGCGCGATCACATGGTCCTGCTGGAGTTCGTGACCGCCGCCGGGATCACTCTCGGCATGGA
CGAGCTGTACAAGTAAAATTATGTCACTATCTAATTATACAGTATTTAGCCATCACAAGACCGTCCAGAC
TAGAGTAGCGCCTAGCTGGCAAAATACTGTGAACC

>SA11_ouNSP1gfp_T6

GGCTTTTTTTTGAAAAGTCTTGTGTTAGCCATGGCTACTTTTAAAGATGCATGCTTTATGGTGAGCAAGG
GCGAGGAGCTGTTCACCGGGGTGGTGCCCATCCTGGTCGAGCTGGACGGCGACGTAAACGGCCACAAGTT
CAGCGTGTCCGGCGAGGGCGAGGGCGATGCCACCTACGGCAAGCTGACCCTGAAGTTCATCTGCACCACC
GGCAAGCTGCCCGTGCCCTGGCCCACCCTCGTGACCACCCTGACCTACGGCGTGCAGTGCTTCAGCCGCT
ACCCCGACCACATGAAGCAGCACGACTTCTTCAAGTCCGCCATGCCCGAAGGCTACGTCCAGGAGCGCAC
CATCTTCTTCAAGGACGACGGCAACTACAAGACCCGCGCCGAGGTGAAGTTCGAGGGCGACACCCTGGTG
AACCGCATCGAGCTGAAGGGCATCGACTTCAAGGAGGACGGCAACATCCTGGGGCACAAGCTGGAGTACA
ACTACAACAGCCACAACGTCTATATCATGGCCGACAAGCAGAAGAACGGCATCAAGGTGAACTTCAAGAT
CCGCCACAACATCGAGGACGGCAGCGTGCAGCTCGCCGACCACTACCAGCAGAACACCCCCATCGGCGAC
GGCCCCGTGCTGCTGCCCGACAACCACTACCTGAGCACCCAGTCCGCCCTGAGCAAAGACCCCAACGAGA
AGCGCGATCACATGGTCCTGCTGGAGTTCGTGACCGCCGCCGGGATCACTCTCGGCATGGACGAGCTGTA
CAAGTAAAATTATGTCACTATCTAATTATACAGTATTTAGCCATCACAAGACCGTCCAGACTAGAGTAGC
GCCTAGCTGGCAAAATACTGTGAACC

Consensus sequences of pruned inserts

>SA11_NSP1gfp_P10 (720 nt pruned)

GGCTTTTTTTTGAAAAGTCTTGTGTTAGCCATGGCTACTTTTAAAGATGCATGCTTTCATTATCGTAGAT
TAACTGCTTTAAATCGGAGATTATGCAACATTGGTGCAAATTCTATTTGGATGCCAGTTCCTGATGCGAA
AATTAAGGGGTGGTGTTTAGAATGTTGTCAAATAGCTGATTTAACCCATTGTTATGGTTGCTCATTGCCG
CATGTTTGCAAATGGTGTGTTCAGAACAGAAGATGCTTCCTTGACAATGAACCTCATTTGCTTAAGCTTA
GAACTGTGAAACATCCAATTACCAAAGACAAATTACAGTGTATCATAGACTTGTACAATATAATATTTCC
AATTAATGATAAAGTAATTAGAAAATTTGAAAGAATGATAAAGCAAAGAGAATGTAGGAATCAATATAAA
ATTGAATGGTATAATCATTTGCTGCTCCCAATTACATTAAATGCTGCTGCATTTAAGTTTGATGAAAATA
ATCTTTATTATGTTTTTGGGTTATATGAGAAATCAGTCAGTGATATATATGCTCCATATAGAATTGTTAA
CTTTATAAATGAATTTGATAAATTATTGCTTGATCATATTAACTTTACAAGAATGTCCAATCTACCAATA
GAGTTGAGAAACCATTACGCAAAGAAATACTTCCAATTATCAAGACTGCCATCATCAAAACTAAAGCAAA
TTTACTTTTCAGATTTTACTAAAGAAACTGTGATTTTTAATACTTATACAAAAACGCCAGGAAGATCAAT
ATACAGAAATGTAACTGAATTTAATTGGAGAGATGAATTGGAGCTTTATTCTGATTTAAAAAATGATAAG
AATAAATTAATTGCTGCAATGATGACGAGTAAGTATACTCGGTTCTATGCTCATGATAATAATTTTGGAA
GGTTGAAAATGACAATATTTGAGTTGGGACATCATTGTCAGCCTAACTACGTGGCATCTAATCACCCAGG
CAATGCTTCCGATATCCAGTACTGTAAATGGTGTAATATAAAATATTTTCTTAGTAAAATTGATTGGCGG
ATTCGTGATATGTATAATTTATTGATGGAATTTATTAAGGATTGTTATAAAAGTAATGTTAACGTTGGAC
ATTGTAGTTCTGTTGAAAACATATATCCTTTAATTAAAAGATTAATTTGGAGTTTGTTTACTAATCACAT
GGTCAAACAATTGAAGAAGTGTTTAATCACATGTCGCCAGTGTCAGTTGAAGGTACGAATGTCATCATGT
TGATTCTTGGATTGAATATTAGTTTGTATAATGAAATTAAGCGCACTTTGAATGTAGATAGCATACCAAT
GGTACTTAATTTAAATGAATTCAGTAGTATAGTTAAATCAATTAGCAGTAAATGGTATAATGTTGATGAA
TTGGATAAATTGCCAATGTCAATAAAATCAACGGAGGAACTGATTGAAATGAAGAATTCTGGAACTTTAA
CTGAAGAATTTGAGCTACTGATCTCCAACTCAGAAGATGACAATGAGTGAAATTATGTCACTATCTAATT
ATACAGTATTTAGCCATCACAAGACCGTCCAGACTAGAGTAGCGCCTAGCTGGCAAAATACTGTGAACC

>SA11_NSP1gfp_P5m (352 nt pruned)

GGCTTTTTTTTGAAAAGTCTTGTGTTAGCCATGGCTACTTTTAAAGATGCATGCTTTCATTATCGTAGAT
TAACTGCTTTAAATCGGAGATTATGCAACATTGGTGCAAATATGGTGA^GGTGAACCGCATCGAGCTGAA
GGGCATCGACTTCAAGGAGGACGGCAACATCCTGGGGCACAAGCTGGAGTACAACTACAACAGCCACAAC
GTCTATATCATGGCCGACAAGCAGAAGAACGGCATCAAGGTGAACTTCAAGATCCGCCACAACATCGAGG
ACGGCAGCGTGCAGCTCGCCGACCACTACCAGCAGAACACCCCCATCGGCGACGGCCCCGTGCTGCTGCC
CGACAACCACTACCTGAGCACCCAGTCCGCCCTGAGCAAAGACCCCAACGAGAAGCGCGATCACATGGTC
CTGCTGGAGTTCGTGACCGCCGCCGGGATCACTCTCGGCATGGACGAGCTGTACAAGTAATCTATTTGGA
TGCCAGTTCCTGATGCGAAAATTAAGGGGTGGTGTTTAGAATGTTGTCAAATAGCTGATTTAACCCATTG
TTATGGTTGCTCATTGCCGCATGTTTGCAAATGGTGTGTTCAGAACAGAAGATGCTTCCTTGACAATGAA

CCTCATTTGCTTAAGCTTAGAACTGTGAAACATCCAATTACCAAAGACAAATTACAGTGTATCATAGACT
TGTACAATATAATATTTCCAATTAATGATAAAGTAATTAGAAAATTTGAAAGAATGATAAAGCAAAGAGA
ATGTAGGAATCAATATAAAATTGAATGGTATAATCATTTGCTGCTCCCAATTACATTAAATGCTGCTGCA
TTTAAGTTTGATGAAAATAATCTTTATTATGTTTTTGGGTTATATGAGAAATCAGTCAGTGATATATATG
CTCCATATAGAATTGTTAACTTTATAAATGAATTTGATAAATTATTGCTTGATCATATTAACTTTACAAG
AATGTCCAATCTACCAATAGAGTTGAGAAACCATTACGCAAAGAAATACTTCCAATTATCAAGACTGCCA
TCATCAAAACTAAAGCAAATTTACTTTTCAGATTTTACTAAAGAAACTGTGATTTTTAATACTTATACAA
AAACGCCAGGAAGATCAATATACAGAAATGTAACTGAATTTAATTGGAGAGATGAATTGGAGCTTTATTC
TGATTTAAAAAATGATAAGAATAAATTAATTGCTGCAATGATGACGAGTAAGTATACTCGGTTCTATGCT
CATGATAATAATTTTGGAAGGTTGAAAATGACAATATTTGAGTTGGGACATCATTGTCAGCCTAACTACG
TGGCATCTAATCACCCAGGCAATGCTTCCGATATCCAGTACTGTAAATGGTGTAATATAAAATATTTTCT
TAGTAAAATTGATTGGCGGATTCGTGATATGTATAATTTATTGATGGAATTTATTAAGGATTGTTATAAA
AGTAATGTTAACGTTGGACATTGTAGTTCTGTTGAAAACATATATCCTTTAATTAAAAGATTAATTTGGA
GTTTGTTTACTAATCACATGGATCAAACAATTGAAGAAGTGTTTAATCACATGTCGCCAGTGTCAGTTGA
AGGTACGAATGTCATCATGTTGATTCTTGGATTGAATATTAGTTTGTATAATGAAATTAAGCGCACTTTG
AATGTAGATAGCATACCAATGGTACTTAATTTAAATGAATTCAGTAGTATAGTTAAATCAATTAGCAGTA
AATGGTATAATGTTGATGAATTGGATAAATTGCCAATGTCAATAAAATCAACGGAGGAACTGATTGAAAT
GAAGAATTCTGGAACTTTAACTGAAGAATTTGAGCTACTGATCTCCAACTCAGAAGATGACAATGAGTGA
AATTATGTCACTATCTAATTATACAGTATTTAGCCATCACAAGACCGTCCAGACTAGAGTAGCGCCTAGC
TGGCAAAATACTGTGAACC

>SA11_NSP1gfp_BamHI_P10B, Clone 1 (696 nt pruned)

GGCTTTTTTTTGAAAAGTCTTGTGTTAGCCATGGCTACTTTTAAAGATGCATGCTTTCATTATCGTAGAT
TAACTGCTTTAAATCGGAGATTATGCAACATTGGTGCAAATATGGTGAGCAAGGGCGAGGAGCTGTTCAC
CGGGGTGGTGCCCATCCTGGTCGAGCTG^AAGGGGTGGTGTTTAGAATGTTGTCAAATAGCTGATTTAAC
CCATTGTTATGGTTGCTCATTGCCGCATGTTTGCAAATGGTGTGTTCAGAACAGAAGATGCTTCCTTGAC
AATGAACCTCATTTGCTTAAGCTTAGAACTGTGAAACATCCAATTACCAAAGACAAATTACAGTGTATCA
TAGACTTGTACAATATAATATTTCCAATTAATGATAAAGTAATTAGAAAATTTGAAAGAATGATAAAGCA
AAGAGAATGTAGGAATCAATATAAAATTGAATGGTATAATCATTTGCTGCTCCCAATTACATTAAATGCT
GCTGCATTTAAGTTTGATGAAAATAATCTTTATTATGTTTTTGGGTTATATGAGAAATCAGTCAGTGATA
TATATGCTCCATATAGAATTGTTAACTTTATAAATGAATTTGATAAATTATTGCTTGATCATATTAACTT
TACAAGAATGTCCAATCTACCAATAGAGTTGAGAAACCATTACGCAAAGAAATACTTCCAATTATCAAGA
CTGCCATCATCAAAACTAAAGCAAATTTACTTTTCAGATTTTACTAAAGAAACTGTGATTTTTAATACTT
ATACAAAAACGCCAGGAAGATCAATATACAGAAATGTAACTGAATTTAATTGGAGAGATGAATTGGAGCT
TTATTCTGATTTAAAAAATGATAAGAATAAATTAATTGCTGCAATGATGACGAGTAAGTATACTCGGTTC
TATGCTCATGATAATAATTTTGGAAGGTTGAAAATGACAATATTTGAGTTGGGACATCATTGTCAGCCTA
ACTACGTGGCATCTAATCACCCAGGCAATGCTTCCGATATCCAGTACTGTAAATGGTGTAATATAAAATA
TTTTCTTAGTAAAATTGATTGGCGGATCCGTGATATGTATAATTTATTGATGGAATTTATTAAGGATTGT
TATAAAAGTAATGTTAACGTTGGACATTGTAGTTCTGTTGAAAACATATATCCTTTAATTAAAAGATTAA
TTTGGAGTTTGTTTACTAATCACATGGATCAAACAATTGAAGAAGTGTTTAATCACATGTCGCCAGTGTC
AGTTGAAGGTACGAATGTCATCATGTTGATTCTTGGATTGAATATTAGTTTGTATAATGAAATTAAGCGC
ACTTTGAATGTAGATAGCATACCAATGGTACTTAATTTAAATGAATTCAGTAGTATAGTTAAATCAATTA
GCAGTAAATGGTATAATGTTGATGAATTGGATAAATTGCCAATGTCAATAAAATCAACGGAGGAACTGAT
TGAAATGAAGAATTCTGGAACTTTAACTGAAGAATTTGAGCTACTGATCTCCAACTCAGAAGATGACAAT
GAGTGAAATTATGTCACTATCTAATTATACAGTATTTAGCCATCACAAGACCGTCCAGACTAGAGTAGCG
CCTAGCTGGCAAAATACTGTGAACC

>SA11_NSP1gfp_BamHI_P10B, Clone 2 (721 nt pruned)

GGCTTTTTTTTGAAAAGTCTTGTGTTAGCCATGGCTACTTTTAAAGATGCATGCTTTCATTATCGTAGAT
TAACTGCTTTAAATCGGAGATTATGCAACATTGGTGCAAATATGGTGAGCAAGGGCGAGGAGCTGTTCAC
CGG^AAGGGGTGGTGTTTAGAATGTTGTCAAATAGCTGATTTAACCCATTGTTATGGTTGCTCATTGCCG

CATGTTTGCAAATGGTGTGTTCAGAACAGAAGATGCTTCCTTGACAATGAACCTCATTTGCTTAAGCTTA
GAACTGTGAAACATCCAATTACCAAAGACAAATTACAGTGTATCATAGACTTGTACAATATAATATTTCC
AATTAATGATAAAGTAATTAGAAAATTTGAAAGAATGATAAAGCAAAGAGAATGTAGGAATCAATATAAA
ATTGAATGGTATAATCATTTGCTGCTCCCAATTACATTAAATGCTGCTGCATTTAAGTTTGATGAAAATA
ATCTTTATTATGTTTTTGGGTTATATGAGAAATCAGTCAGTGATATATATGCTCCATATAGAATTGTTAA
CTTTATAAATGAATTTGATAAATTATTGCTTGATCATATTAACTTTACAAGAATGTCCAATCTACCAATA
GAGTTGAGAAACCATTACGCAAAGAAATACTTCCAATTATCAAGACTGCCATCATCAAAACTAAAGCAAA
TTTACTTTTCAGATTTTACTAAAGAAACTGTGATTTTTAATACTTATACAAAAACGCCAGGAAGATCAAT
ATACAGAAATGTAACTGAATTTAATTGGAGAGATGAATTGGAGCTTTATTCTGATTTAAAAAATGATAAG
AATAAATTAATTGCTGCAATGATGACGAGTAAGTATACTCGGTTCTATGCTCATGATAATAATTTTGGAA
GGTTGAAAATGACAATATTTGAGTTGGGACATCATTGTCAGCCTAACTACGTGGCATCTAATCACCCAGG
CAATGCTTCCGATATCCAGTACTGTAAATGGTGTAATATAAAATATTTTCTTAGTAAAATTGATTGGCGG
ATCCGTGATATGTATAATTTATTGATGGAATTTATTAAGGATTGTTATAAAAGTAATGTTAACGTTGGAC
ATTGTAGTTCTGTTGAAAACATATATCCTTTAATTAAAAGATTAATTTGGAGTTTGTTTACTAATCACAT
GGATCAAACAATTGAAGAAGTGTTTAATCACATGTCGCCAGTGTCAGTTGAAGGTACGAATGTCATCATG
TTGATTCTTGGATTGAATATTAGTTTGTATAATGAAATTAAGCGCACTTTGAATGTAGATAGCATACCAA
TGGTACTTAATTTAAATGAATTCAGTAGTATAGTTAAATCAATTAGCAGTAAATGGTATAATGTTGATGA
ATTGGATAAATTGCCAATGTCAATAAAATCAACGGAGGAACTGATTGAAATGAAGAATTCTGGAACTTTA
ACTGAAGAATTTGAGCTACTGATCTCCAACTCAGAAGATGACAATGAGTGAAATTATGTCACTATCTAAT
TATACAGTATTTAGCCATCACAAGACCGTCCAGACTAGAGTAGCGCCTAGCTGGCAAAATACTGTGAACC

>SA11_ouNSP1gfp_P10, Clone 1 (297 nt pruned)

GGCTTTTTTTTGAAAAGTCTTGTGTTAGCCATGGCTACTTTTAAAGATGCATGCTTTCATTATCGTAGAT
TAACTGCTTTAAATCGGAGATTATGCAACATTGGTGCAAAT^TTCTTCAAGGACGACGGCAACTACAAGA
CCCGCGCCGAGGTGAAGTTCGAGGGCGACACCCTGGTGAACCGCATCGAGCTGAAGGGCATCGACTTCAA
GGAGGACGGCAACATCCTGGGGCACAAGCTGGAGTACAACTACAACAGCCACAACGTCTATATCATGGCC
GACAAGCAGAAGAACGGCATCAAGGTGAACTTCAAGATCCGCCACAACATCGAGGACGGCAGCGTGCAGC
TCGCCGACCACTACCAGCAGAACACCCCCATCGGCGACGGCCCCGTGCTGCTGCCCGACAACCACTACCT
GAGCACCCAGTCCGCCCTGAGCAAAGACCCCAACGAGAAGCGCGATCACATGGTCCTGCTGGAGTTCGTG
ACCGCCGCCGGGATCACTCTCGGCATGGACGAGCTGTACAAGTAAAATTATGTCACTATCTAATTATACA
GTATTTAGCCATCACAAGACCGTCCAGACTAGAGTAGCGCCTAGCTGGCAAAATACTGTGAACC

>SA11_ouNSP1gfp_P10, Clone 2 (300 nt pruned)

GGCTTTTTTTTGAAAAGTCTTGTGTTAGCCATGGCTACTTTTAAAGATGCATGCTTTCATTATCGTAGAT
TAACTGCTTTAAATCGGAGATTATGCAACATTGGTGCAAAT^TTCAAGGACGACGGCAACTACAAGACCC
GCGCCGAGGTGAAGTTCGAGGGCGACACCCTGGTGAACCGCATCGAGCTGAAGGGCATCGACTTCAAGGA
GGACGGCAACATCCTGGGGCACAAGCTGGAGTACAACTACAACAGCCACAACGTCTATATCATGGCCGAC
AAGCAGAAGAACGGCATCAAGGTGAACTTCAAGATCCGCCACAACATCGAGGACGGCAGCGTGCAGCTCG
CCGACCACTACCAGCAGAACACCCCCATCGGCGACGGCCCCGTGCTGCTGCCCGACAACCACTACCTGAG
CACCCAGTCCGCCCTGAGCAAAGACCCCAACGAGAAGCGCGATCACATGGTCCTGCTGGAGTTCGTGACC
GCCGCCGGGATCACTCTCGGCATGGACGAGCTGTACAAGTAAAATTATGTCACTATCTAATTATACAGTA
TTTAGCCATCACAAGACCGTCCAGACTAGAGTAGCGCCTAGCTGGCAAAATACTGTGAACC

>SA11_ouNSP1gfp_P10, Clone 3 (265 nt pruned)

GGCTTTTTTTTGAAAAGTCTTGTGTTAGCCATGGCTACTTTTAAAGATGCATGCTTTCATTATCGTAGAT
TAACTGCTTTAAATCGGAGATTATGCAACATTGGTGCAAAT^TGCCCGAAGGCTACGTCCAGGAGCGCAC
CATCTTCTTCAAGGACGACGGCAACTACAAGACCCGCGCCGAGGTGAAGTTCGAGGGCGACACCCTGGTG
AACCGCATCGAGCTGAAGGGCATCGACTTCAAGGAGGACGGCAACATCCTGGGGCACAAGCTGGAGTACA
ACTACAACAGCCACAACGTCTATATCATGGCCGACAAGCAGAAGAACGGCATCAAGGTGAACTTCAAGAT
CCGCCACAACATCGAGGACGGCAGCGTGCAGCTCGCCGACCACTACCAGCAGAACACCCCCATCGGCGAC
GGCCCCGTGCTGCTGCCCGACAACCACTACCTGAGCACCCAGTCCGCCCTGAGCAAAGACCCCAACGAGA

AGCGCGATCACATGGTCCTGCTGGAGTTCGTGACCGCCGCCGGGATCACTCTCGGCATGGACGAGCTGTA
CAAGTAAAATTATGTCACTATCTAATTATACAGTATTTAGCCATCACAAGACCGTCCAGACTAGAGTAGC
GCCTAGCTGGCAAAATACTGTGAACC

>SA11_ouNSP1gfp_T4_P10 (286 nt pruned)

GGCTTTTTTTTGAAAAGTCTTGTGTTAGCCATGGCTACTTTTAAAGATGCATGCTTTCATTATCGTAGAT
TAACTATGGTGAGCAAGGGCGAGG^GGACGACGGCAACTACAAGACCCGCGCCGAGGTGAAGTTCGAGGG
CGACACCCTGGTGAACCGCATCGAGCTGAAGGGCATCGACTTCAAGGAGGACGGCAACATCCTGGGGCAC
AAGCTGGAGTACAACTACAACAGCCACAACGTCTATATCATGGCCGACAAGCAGAAGAACGGCATCAAGG
TGAACTTCAAGATCCGCCACAACATCGAGGACGGCAGCGTGCAGCTCGCCGACCACTACCAGCAGAACAC
CCCCATCGGCGACGGCCCCGTGCTGCTGCCCGACAACCACTACCTGAGCACCCAGTCCGCCCTGAGCAAA
GACCCCAACGAGAAGCGCGATCACATGGTCCTGCTGGAGTTCGTGACCGCCGCCGGGATCACTCTCGGCA
TGGACGAGCTGTACAAGTAAAATTATGTCACTATCTAATTATACAGTATTTAGCCATCACAAGACCGTCC
AGACTAGAGTAGCGCCTAGCTGGCAAAATACTGTGAACC

>SA11_ouNSP1gfp_T5_P10 (350 nt pruned)

GGCTTTTTTTTGAAAAGTCTTGTGTTAGCCATGGCTACTTTTAAAGATGCATGCTTTCATTATCGTATGG
TGAGCAAGGGCGAGGAGCTGTTC^GAAGGGCATCGACTTCAAGGAGGACGGCAACATCCTGGGGCACAAG
CTGGAGTACAACTACAACAGCCACAACGTCTATATCATGGCCGACAAGCAGAAGAACGGCATCAAGGTGA
ACTTCAAGATCCGCCACAACATCGAGGACGGCAGCGTGCAGCTCGCCGACCACTACCAGCAGAACACCCC
CATCGGCGACGGCCCCGTGCTGCTGCCCGACAACCACTACCTGAGCACCCAGTCCGCCCTGAGCAAAGAC
CCCAACGAGAAGCGCGATCACATGGTCCTGCTGGAGTTCGTGACCGCCGCCGGGATCACTCTCGGCATGG
ACGAGCTGTACAAGTAAAATTATGTCACTATCTAATTATACAGTATTTAGCCATCACAAGACCGTCCAGA
CTAGAGTAGCGCCTAGCTGGCAAAATACTGTGAACC

>SA11_ouNSP1gfp_T6_P10 (283 nt pruned)

GGCTTTTTTTTGAAAAGTCTTGTGTTAGCCATGGCTACTTTTAAAGATGCATGCTTT^AGGAGCGCACCA
TCTTCTTCAAGGACGACGGCAACTACAAGACCCGCGCCGAGGTGAAGTTCGAGGGCGACACCCTGGTGAA
CCGCATCGAGCTGAAGGGCATCGACTTCAAGGAGGACGGCAACATCCTGGGGCACAAGCTGGAGTACAAC
TACAACAGCCACAACGTCTATATCATGGCCGACAAGCAGAAGAACGGCATCAAGGTGAACTTCAAGATCC
GCCACAACATCGAGGACGGCAGCGTGCAGCTCGCCGACCACTACCAGCAGAACACCCCCATCGGCGACGG
CCCCGTGCTGCTGCCCGACAACCACTACCTGAGCACCCAGTCCGCCCTGAGCAAAGACCCCAACGAGAAG
CGCGATCACATGGTCCTGCTGGAGTTCGTGACCGCCGCCGGGATCACTCTCGGCATGGACGAGCTGTACA
AGTAAAATTATGTCACTATCTAATTATACAGTATTTAGCCATCACAAGACCGTCCAGACTAGAGTAGCGC
CTAGCTGGCAAAATACTGTGAACC
